# Chronically elevated branched chain amino acid levels are pro-arrhythmic

**DOI:** 10.1093/cvr/cvab207

**Published:** 2021-06-17

**Authors:** Vincent Portero, Thomas Nicol, Svitlana Podliesna, Gerard A Marchal, Antonius Baartscheer, Simona Casini, Rafik Tadros, Jorien L Treur, Michael W T Tanck, I Jane Cox, Fay Probert, Tertius A Hough, Sara Falcone, Leander Beekman, Martina Müller-Nurasyid, Gabi Kastenmüller, Christian Gieger, Annette Peters, Stefan Kääb, Moritz F Sinner, Andrew Blease, Arie O Verkerk, Connie R Bezzina, Paul K Potter, Carol Ann Remme

**Affiliations:** Heart Center, Department of Clinical and Experimental Cardiology, Amsterdam UMC, Location AMC, Room K2-104.2, Meibergdreef 9, PO Box 22700, 1100 DE Amsterdam, The Netherlands; Mammalian Genetics Unit, MRC Harwell Institute, Harwell, Oxfordshire, UK; Heart Center, Department of Clinical and Experimental Cardiology, Amsterdam UMC, Location AMC, Room K2-104.2, Meibergdreef 9, PO Box 22700, 1100 DE Amsterdam, The Netherlands; Heart Center, Department of Clinical and Experimental Cardiology, Amsterdam UMC, Location AMC, Room K2-104.2, Meibergdreef 9, PO Box 22700, 1100 DE Amsterdam, The Netherlands; Heart Center, Department of Clinical and Experimental Cardiology, Amsterdam UMC, Location AMC, Room K2-104.2, Meibergdreef 9, PO Box 22700, 1100 DE Amsterdam, The Netherlands; Heart Center, Department of Clinical and Experimental Cardiology, Amsterdam UMC, Location AMC, Room K2-104.2, Meibergdreef 9, PO Box 22700, 1100 DE Amsterdam, The Netherlands; Cardiovascular Genetics Center, Montreal Heart Institute and Faculty of Medicine, Université de Montréal, Montreal, Canada; Department of Psychiatry, Amsterdam UMC, Location AMC, Amsterdam, The Netherlands; Amsterdam UMC, University of Amsterdam, Department of Epidemiology and Data Science, Amsterdam Public Health (APH), The Netherlands; Institute of Hepatology London, Foundation for Liver Research, London, UK; Faculty of Life Sciences & Medicine, Kings College, London, UK; Department of Chemistry, University of Oxford, Oxford, UK; Mammalian Genetics Unit, MRC Harwell Institute, Harwell, Oxfordshire, UK; Mammalian Genetics Unit, MRC Harwell Institute, Harwell, Oxfordshire, UK; Heart Center, Department of Clinical and Experimental Cardiology, Amsterdam UMC, Location AMC, Room K2-104.2, Meibergdreef 9, PO Box 22700, 1100 DE Amsterdam, The Netherlands; Research Unit of Molecular Epidemiology, Helmholtz Zentrum München, German Research Center for Environmental Health, Neuherberg, Germany; IBE, Faculty of Medicine, Ludwig Maximilian’s University (LMU) Munich, Munich, Germany; Institute of Medical Biostatistics, Epidemiology and Informatics (IMBEI), University Medical Center, Johannes Gutenberg University, Mainz, Germany; Institute of Bioinformatics and Systems Biology, Helmholtz Zentrum München, German Research Center for Environmental Health, Neuherberg, Germany; German Center for Diabetes Research (DZD), Neuherberg, Germany; Research Unit of Molecular Epidemiology, Helmholtz Zentrum München, German Research Center for Environmental Health, Neuherberg, Germany; German Center for Diabetes Research (DZD), Neuherberg, Germany; Institute of Human Genetics, Helmholtz Zentrum München, German Research Center for Environmental Health, Neuherberg, Germany; Institute of Epidemiology II, Helmholtz Zentrum München, German Research Center for Environmental Health, Neuherberg, Germany; German Centre for Cardiovascular Research (DZHK), Partner Site: Munich Heart Alliance, Munich, Germany; Institute of Epidemiology II, Helmholtz Zentrum München, German Research Center for Environmental Health, Neuherberg, Germany; German Centre for Cardiovascular Research (DZHK), Partner Site: Munich Heart Alliance, Munich, Germany; German Centre for Cardiovascular Research (DZHK), Partner Site: Munich Heart Alliance, Munich, Germany; Department of Medicine I (Cardiology), University Hospital, LMU Munich, Munich, Germany; German Centre for Cardiovascular Research (DZHK), Partner Site: Munich Heart Alliance, Munich, Germany; Department of Medicine I (Cardiology), University Hospital, LMU Munich, Munich, Germany; Mammalian Genetics Unit, MRC Harwell Institute, Harwell, Oxfordshire, UK; Heart Center, Department of Clinical and Experimental Cardiology, Amsterdam UMC, Location AMC, Room K2-104.2, Meibergdreef 9, PO Box 22700, 1100 DE Amsterdam, The Netherlands; Heart Center, Department of Clinical and Experimental Cardiology, Amsterdam UMC, Location AMC, Room K2-104.2, Meibergdreef 9, PO Box 22700, 1100 DE Amsterdam, The Netherlands; Department of Biological and Medical Sciences, Faculty of Health and Life Sciences, Oxford Brookes University, Oxford, UK; Heart Center, Department of Clinical and Experimental Cardiology, Amsterdam UMC, Location AMC, Room K2-104.2, Meibergdreef 9, PO Box 22700, 1100 DE Amsterdam, The Netherlands

**Keywords:** Arrhythmia, Electrophysiology, Sudden death, Metabolism, BCAA

## Abstract

**Aims:**

Cardiac arrhythmias comprise a major health and economic burden and are associated with significant morbidity and mortality, including cardiac failure, stroke, and sudden cardiac death (SCD). Development of efficient preventive and therapeutic strategies is hampered by incomplete knowledge of disease mechanisms and pathways. Our aim is to identify novel mechanisms underlying cardiac arrhythmia and SCD using an unbiased approach.

**Methods and results:**

We employed a phenotype-driven *N*-ethyl-*N*-nitrosourea mutagenesis screen and identified a mouse line with a high incidence of sudden death at young age (6–9 weeks) in the absence of prior symptoms. Affected mice were found to be homozygous for the nonsense mutation *Bcat2*^p.Q300*/p.Q300*^ in the *Bcat2* gene encoding branched chain amino acid transaminase 2. At the age of 4–5 weeks, *Bcat2*^p.Q300*/p.Q300*^ mice displayed drastic increase of plasma levels of branch chain amino acids (BCAAs—leucine, isoleucine, valine) due to the incomplete catabolism of BCAAs, in addition to inducible arrhythmias *ex vivo* as well as cardiac conduction and repolarization disturbances. In line with these findings, plasma BCAA levels were positively correlated to electrocardiogram indices of conduction and repolarization in the German community-based KORA F4 Study. Isolated cardiomyocytes from *Bcat2*^p.Q300*/p.Q300*^ mice revealed action potential (AP) prolongation, pro-arrhythmic events (early and late afterdepolarizations, triggered APs), and dysregulated calcium homeostasis. Incubation of human pluripotent stem cell-derived cardiomyocytes with elevated concentration of BCAAs induced similar calcium dysregulation and pro-arrhythmic events which were prevented by rapamycin, demonstrating the crucial involvement of mTOR pathway activation.

**Conclusions:**

Our findings identify for the first time a causative link between elevated BCAAs and arrhythmia, which has implications for arrhythmogenesis in conditions associated with BCAA metabolism dysregulation such as diabetes, metabolic syndrome, and heart failure.

## Graphical Abstract

**Graphical Abstract:**
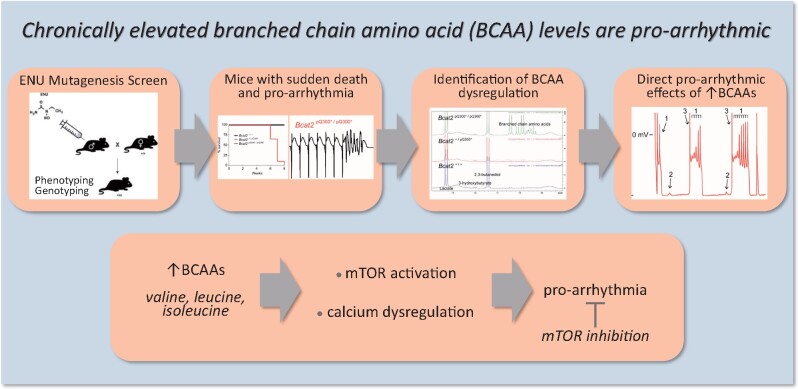

## 1. Introduction

Cardiac arrhythmias comprise a major health and economic burden and are associated with significant morbidity and mortality, including cardiac failure, stroke, and sudden cardiac death (SCD). SCD remains a leading cause of mortality in the Western world, accounting for up to 20% of all natural deaths, and up to 50% of all cardiovascular deaths.^1^ Arrhythmias typically occur in the setting of an underlying pathology, including myocardial ischaemia, structural derangements, and co-morbidities such as hypertension.^1,2^ Furthermore, patients suffering from metabolic disorders (diabetes, obesity) and heart failure are at increased risk for arrhythmias and SCD.^3–8^ Despite decades of research, few anti-arrhythmic therapeutic options exist due to the complexity of underlying pathologies. Development of efficient preventive and therapeutic strategies is essential but is as yet hampered by incomplete knowledge of disease mechanisms.

Over the last decade, we and others have employed several novel approaches to identify new disease mechanisms and pathways regulating cardiac electrical (dys)function, including genomic studies in rodents.^9,10^ Phenotype driven screens have been very successful in identifying novel genes and alleles associated with disease, and resolving gene function.^11,12^ In *N*-ethyl-*N*-nitrosourea (ENU) mutagenesis screens, pedigrees of mice with randomly induced point mutations are screened for a range of phenotypes and affected mice are subsequently used to map and clone the causative allele.^13^ Employing such a phenotype-driven ENU screen, we here identified a mutant mouse line presenting with sudden death and a homozygous nonsense mutation in the *Bcat2* gene. Affected mice displayed a drastic increase of plasma branched chain amino acid (BCAA—valine, isoleucine, leucine) levels due to the incomplete catabolism of BCAAs, enhanced arrhythmia susceptibility, cardiac conduction and repolarization disturbances, and excessive pro-arrhythmic intracellular calcium dysregulation in isolated cardiomyocytes. In support of a role for BCAAs in modulation of cardiac electrical function, plasma BCAA levels were positively correlated to electrocardiogram (ECG) indices of conduction and repolarization in individuals from the general population.^14^ Studies in human pluripotent stem cell-derived cardiomyocytes (hPSC-CMs) furthermore confirmed a direct pro-arrhythmic effect of elevated BCAAs and demonstrated the crucial involvement of the mammalian target of rapamycin (mTOR) activation in mediating these effects. Our findings thus demonstrate a causative link between elevated BCAAs and arrhythmia, which has implications for furthering our understanding of mechanisms underlying lethal arrhythmias, particularly those occurring in disease states associated with elevated BCAA levels such as diabetes and metabolic syndrome.^4–6,14–17^

## 2. Materials and methods

### 2.1 Mice

All animals were housed and maintained in the Mary Lyon Centre at MRC Harwell, under specific pathogen-free conditions in individually ventilated cages, with environmental conditions as outlined in the Home Office Code of Practice. Home Office ethical approval was granted under project license 30/3070 and mice were euthanized by Home Office Schedule 1 methods. All experiments involving mice conformed to governmental and institutional guidelines for animal use in research and were performed with approval of the Animal Experimental Committee of the Academic Medical Center, Amsterdam (license AVD1180020184986). For ECG measurements, general anaesthesia was induced with 4.0% isoflurane and maintained at 0.8–1.2% isoflurane in oxygen. Euthanasia was performed by 100% CO_2_ administration (20% v/v per minute), followed by cervical dislocation.

### 2.2 Generation of mutagenized pedigrees

The details of the ENU program have been described previously.^11^ Briefly, C57BL/6J male mice (G_0_) were treated with ENU doses of 1 × 120 mg kg^–1^, and then 2 × 100 mg kg^–1^with a week between each dose. The mice were then bred with wild-type ‘sighted C3H’ (C3H.Pde6b+) females.^12^ The resulting G_1_ males were bred with wild-type C3H.Pde6b+ females to produce G_2_ females, which were backcrossed to their G_1_ fathers to generate G_3_ offspring. The G_3_ mating scheme results on average in 1 in 8 G_3_ mice homozygous for any particular ENU-induced mutation. Binomial probability calculations indicate that a cohort of 50 single sex mice will yield 4 homozygotes with a probability of ∼85% and 5 with a probability of ∼75%. Initial identification of the sudden death phenotype was in G3 mice (batch A) but subsequent studies employed mice backcrossed ten generations to the C3H.Pde6b+ background (batch B). Measurements and experiments were performed in mice of either sex at the age of 4–5 weeks, unless otherwise specified (as further specified in the Supplementary material online).

### 2.3 Mapping and DNA sequencing

DNA from affected mice and littermates was prepared from ear biopsies and used for linkage mapping utilizing the Illumina GoldenGate Mouse Medium Density Linkage Panel (Gen-Probe Life Sciences Ltd, UK). DNA from the founder G_1_ mouse was prepared for whole-genome sequencing (WGS) using the Nucleon BACC2 Genomic DNA Extraction System (GE Healthcare Life Sciences). After library generation, a single lane of paired-end sequencing (100 nt) was performed employing the Illumina HiSeq platform (Oxford Genomics Centre, Wellcome Trust Centre for Human Genetics) and analysed as previously described.^11^ Genetic variants identified by WGS were confirmed by pyrosequencing (see Supplementary material online).

### 2.4 NMR methodology and biochemical screen

Because of the severe nature of the phenotype and risk of early death, the phenotyping of this line was limited primarily to post mortem analysis. The function of the *Bcat2* gene suggested the possibility of abnormal levels of metabolites such as branched chain amino acids. To prevent the unnecessary death of mice, urine and plasma of male and female mice aged between 4 and 5 weeks of age were analysed by NMR. Details on urine and plasma sample preparation, NMR analysis, and estimation of the valine and xleucine (leucine and isoleucine combined) concentrations from the NMR spectra are provided in the Supplementary material online. Further analysis of plasma was carried out by a clinical chemistry analysis of terminal plasma samples; further details are provided in the Supplementary material online, *Table S1*.

### 2.5 Western blot analysis of WT and p.Q300* BCAT2 protein expression

Full length cDNA clone of *BCAT2* (Dharmacon) was ligated into the pCMV6-AN-Myc vector (Origene), and the C1121T mutation was introduced by Q5 site-directed mutagenesis. Plasmids were transfected into HEK293T cells followed by western blot analysis as detailed in the Supplementary material online.

### 2.6 mTOR and P-mTOR (Ser2448) protein expression in mouse hearts

Hearts from *Bcat2*^+/+^ and *Bcat2*^pQ330*/pQ330*^ homozygous males were harvested between 5 and 6 weeks of age and snap frozen in liquid nitrogen. Proteins were extracted from cardiac apex in RIPA buffer and 40 µg of protein was loaded for each sample. Primary antibodies used were mTOR 1:1000 (Cell signalling technology, ref: 2972); mTOR-P-ser2448 1:1000 (Cell signalling technology, ref: 2971), GAPDH 1:10 000 (Fitzgerald, ref: 10R-G109A), and goat anti-rabbit and anti-mouse horseradish peroxidase–conjugated secondary antibodies from GE Healthcare Life Science (ref: NA9310V and NA9340V, respectively; 1:10 000). For each blot, total mTOR and mTOR-P-ser2448 protein expression protein were normalized to the GAPDH signal. Full details are provided in the Supplementary material online.

### 2.7 Cardiac structural abnormalities

To assess the presence of cardiac hypertrophy, heart weight/body weight, and heart weight/tibia length ratios were calculated. Histological assessment of cardiac tissue was carried out on haematoxylin and eosin stained tissue obtained from 6-week-old G3 mice. Both female and male mice were included in the quantification.

### 2.8 ECG measurements, arrhythmia inducibility testing, and optical mapping

Surface ECG measurements were performed in mice anaesthetized by isoflurane inhalation using the Powerlab acquisition system (ADInstruments). Cardiac arrhythmia inducibility was assessed in isolated, Langendorff-perfused hearts. Atria and ventricles were stimulated at a basic cycle length (BCL) of 120 ms and arrhythmia inducibility was evaluated using up to three extrastimuli (S1-S2-S3) and burst pacing. Optical mapping was carried out in Langendorff-perfused hearts paced at a BCL of 120 ms from the centre of the ventricular epicardial surface. Optical action potentials (APs) were analysed, and local activation defined as the maximum positive slope of the AP was calculated using custom software. Measured local activation times were used to construct ventricular activation maps and calculate conduction velocity in longitudinal and transversal directions. For all electrophysiological measurements, both female and male mice (aged 4–5 weeks) were used. More details can be found in the Supplementary material online.

### 2.9 Human pluripotent stem cell-derived cardiomyocytes

Cor4.U control hPSC-CMs were purchased from Ncardia (Leiden, The Netherlands) and were plated on gelatin (0.1% diluted in PBS) coated coverslips at a density of 5000 cells per coverslip following manufacturer’s instructions. The generation and use of these cells were conform to the declaration of Helsinki. Cells were first plated in complete culture medium (Ncardia) for 24 h and then cultured for 3–4 days in PMC medium (Ncardia) with modified branched chain amino acid (BCAA) concentration. BCAA concentrations in control condition were normalized to 800 nmol/L and supplemented to similar concentrations as quantified by NMR in plasma samples from *Bcat2*^p.^^Q300*/p.Q300*^ mice (i.e. valine: 10 mmol/L; leucine: 7.5 mmol/L; isoleucine: 4.5 mmol/L). BCAAs were purchased from Sigma-Aldrich and diluted in PMC medium. Rapamycin (Sigma-Aldrich) was dissolved in dimethyl sulfoxide (DMSO) at a stock concentration of 20 mmol/L. For experiments, Rapamycin was used at a concentration of 500 nmol/L diluted in PMC medium (final DMSO concentration 0.0025%).

### 2.10 Action potential measurements

Details on isolation of ventricular mouse cardiomyocytes and AP measurements are provided in the Supplementary material online. In short, APs were measured at 36°C and were elicited at 2 Hz in mouse cardiomyocytes and 1 Hz in hPSC-CMs. Typically, hPSC-CMs have a small or even complete lack of the inward rectifying potassium current (*I*_K1_). Consequently, hPSC-CMs have a depolarized resting membrane potential (RMP) and are frequently spontaneously active.^18^ To overcome these conditions, we injected an *in silico I*_K1_ with kinetics of Kir2.1 channel through dynamic clamp,^19^ as previously described and validated.^20,21^ Consequently, cells became quiescent with an RMP of around −82 mV. We analysed RMP, AP amplitude (APA), maximal AP upstroke velocity (*V*_max_), and APD at 20%, 50%, and 90% repolarization (APD_20_, APD_50_, and APD_90_, respectively). The fast-pacing protocol used to count and quantify early after depolarizations (EADs), delayed after depolarizations (DADs), and triggered APs (TAPs) consisted of 20 pulses at a frequency of 5 Hz followed by a 10 s pause. Average results were obtained from five consecutive traces.

### 2.11 Intracellular calcium measurements

Calcium transients were measured at 37°C in Tyrode solution (see Supplementary material online) in isolated myocytes stimulated at 6 Hz using the fluorescent probe Indo-1 as described previously.^22^ Calcium after-transients were elicited in myocytes stimulated at 6 Hz in which stimulation was stopped and spontaneous activity was recorded for 10 s.^23^ Rapid cooling, which causes complete depletion of calcium from the sarcoplasmic reticulum (SR), was used to estimate SR calcium content as described previously.^23^ Calcium transient decay time was determined using a mono-exponential fit.

### 2.12 Association between plasma BCAA levels and ECG measures in the KORA F4 Study

The community-based KORA Study (Cooperative Health Research in the Augsburg Region) was conducted in Augsburg, Southern Germany.^14^ The KORA F4 Study recruited 3080 participants between 2006 and 2008. All study participants provided written informed consent, and the study was approved by the Bayerische Landesärztekammer and is conform to the declaration of Helsinki. Peripheral blood from fasting individuals was drawn for biomarker analyses. In all participants, a standard 12-lead ECG was obtained after 10 minutes rest in supine position. All participants underwent metabolic profiling using the Biocrates AbsoluteIDQp150 kit (Biocates Life Sciences AG, Innsbruck, Austria). For the current analysis, µmol/L concentrations of the BCAAs valine and xleucine (i.e. the combination of leucine and isoleucine) were used. Further details on data collection and exclusion criteria are provided in the Supplementary material online. Overall, 776 participants were excluded, leaving 2304 individuals in the final data set.

### 2.13 Statistical analysis

No statistical methods were used to predetermine sample size. Differences between two unpaired groups were assessed using two-tailed *t*-tests when following a normal distribution and Mann-Whitney Rank sum test if normality test failed. Differences comparing three groups, when normally distributed were assessed using one-way analysis of variance (ANOVA) with Student–Newman–Keuls *posthoc* test and using one-way ANOVA on ranks (Dunn’s method *posthoc*) when data were not normally distributed. All statistical tests were performed using sigma stat 3.5 software (Systat Software, Inc). Variability in all plots and graphs is presented as the S.E.M. All *P* < 0.05 were considered to be significant. *P < 0.05; **P ≤ 0.01; ^#^*P* ≤ 0.001. Summary statistics are depicted in Supplementary material online, *Tables S2*–*S9*. To determine a relation between ECG measures and BCAAs in the KORA population, linear regression models were fitted, adjusting for sex. Multiplicative interaction terms showed no statistical interactions with age or sex, and interaction terms were removed from the final models. All statistical analyses in KORA were performed using STATA 12.0 (StataCorp LP, College Station, TX, USA).

## 3. Results

### 3.1 Sudden death phenotype in homozygous Bcat2^p.Q300^^*^^/p.Q300*^ mice

**Figure 1:**
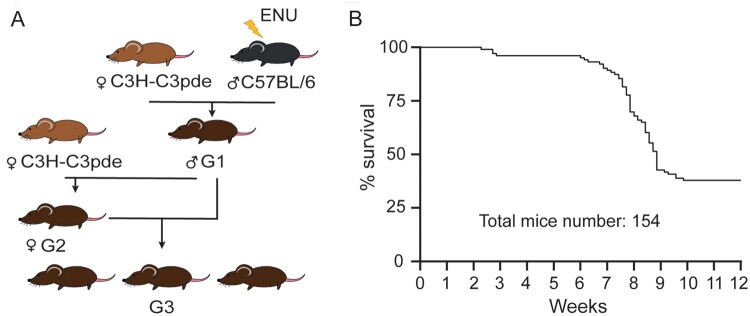
ENU mutagenesis-induced sudden death phenotype in young mice. (*A*) Breeding scheme used to produce G3 phenotyping cohorts. C57BL/6J males were mutagenized with ENU and crossed to C3H.Pde6b+ females. G1 males were then crossed again to C3H.Pde6b+ females, producing a G2 generation. Finally, G2 females were crossed to the G1 male parent to produce a G3 phenotyping cohort. (*B*) Mortality curve of the initial G3 phenotyping pedigrees (batch A; *n* = 154 mice) demonstrating a clear early death phenotype between 6 and 9 weeks of age.

**Figure 2:**
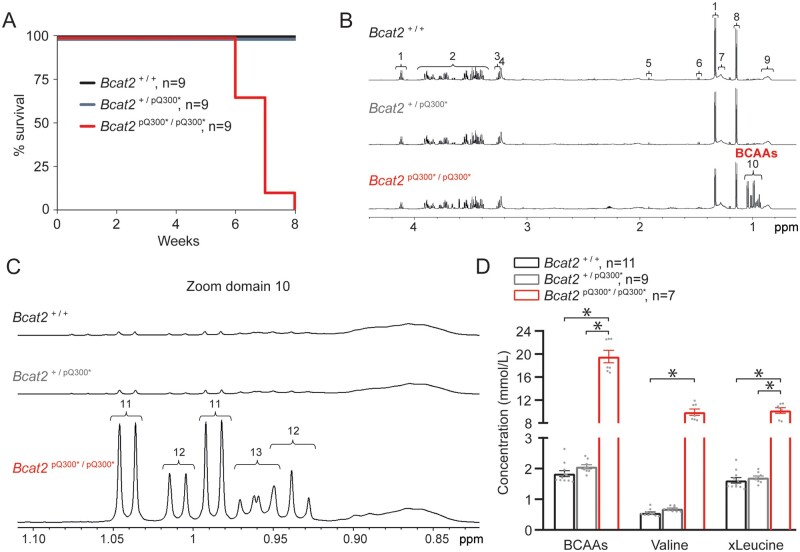
Homozygous *Bcat2*^p.Q300*/p.Q300*^ mice display sudden death and increased plasma BCAA levels at young age. (*A*) Mortality curve displaying death of all homozygous *Bcat2*^p.Q300*/p.Q300*^ mice (batch B) by the age of 8 weeks. (*B*) Illustrative water suppressed spin-echo proton NMR spectra of free-fed plasma from *Bcat2*^+/+^, *Bcat2*^+/p.Q300*^, and *Bcat2*^p.Q300*/p.Q300*^ mice at 5 weeks of age. The NMR spectra were plotted such that the αC1 proton of glucose at 5.23 ppm (not shown) was of similar intensity in each data set. The prominent metabolites shown in the expanded region are assigned to: (1) lactate, (2) glucose, (3) including β-glucose, (4) choline-containing compounds, (5) acetate, (6) alanine, (7) mobile lipid CH_2_, (8) 2,3-butanediol, (9) mobile lipid CH_3_, (10) branched chain amino acids (BCAAs). (*C*) NMR spectra from *Bcat2*^+/+^, *Bcat2*^+/p.Q300*^, *Bcat2*^p.Q300*/p.Q300*^ further expanded (zoom domain 10) to illustrate the branched chain amino acid -CH_3_ region with metabolites assigned to (11) valine, (12) isoleucine, (13) leucine. (*D*) BCAA plasmatic quantification in *Bcat2*^+/+^ (*n* = 11), *Bcat2*^+/p.Q300*^ (*n* = 9) and *Bcat2*^p.Q300*/p.Q300*^ (*n* = 7) mice. *P < 0.05, one-way ANOVA on ranks with *post hoc* Dunn’s method.

Following ENU mutagenesis of C57BL/6J male mice (G_0_) and successive rounds of breeding with C3H.Pde6b+ females (*Figure 1A*), G3 cohorts of mutagenized animals were produced for longitudinal phenotyping studies as part of the Harwell Ageing Screen.^11^ Mice from a single pedigree, consisting of mixed background G3 mice died suddenly with no obvious cause and in the absence of preceding symptoms such as tremoring or gait abnormalities, between 6 and 9 weeks of age (batch A; *Figure 1B*). Mapping was performed on the basis that homozygous ENU-induced mutations must lay in a region of C57BL/6J homogeneity and a region was identified on chromosome 7 spanning from 28.8 Mb to 56.1 Mb. WGS of the G1 founder male revealed two high confidence coding mutations within the mapped region, *Bcat2^C1121T^* and *Siglech^C751T^* resulting in a Q300* and Q219* nonsense mutation, respectively. Pyrosequencing confirmed that both mutations were present in individuals affected by the sudden death phenotype but absent from unaffected controls. Further breeding eliminated *Siglech* as a candidate by narrowing the mapped region and confirmed that all affected mice suffering sudden death were homozygous for the nonsense mutation *Bcat2^C1121T^* in the gene encoding branched chain aminotransferase 2 (Supplementary material online, *Figure S1*). No heterozygous *Bcat2^C1121T^* or wild-type mice succumbed to sudden death before the age of eight weeks and no homozygous mice lived beyond this age (batch B; *Figure 2A*). The *Bcat2^C1121T^* mutation results in an early stop (Q300*) and consequently a truncated BCAT2 protein lacking the CXXC motif essential for enzyme catalytic activity and substrate orientation (Supplementary material online, *Figure S2*). Plasma clinical chemistry revealed increased levels of triglycerides, fatty acids, and glycerol, as well as LDL in homozygous *Bcat2*^p.Q300*/p.Q300*^ mice and intermediate levels in heterozygous *Bcat2*^+/p.Q300*^ mice (Supplementary material online, *Figure S3* and *Table S3*). Homozygous *Bcat2*^p.Q300*/p.Q300*^ mice exhibited increased potassium but decreased chloride concentration in plasma, and increased iron levels. Whilst significant, none of these data provide a clear explanation for the sudden death phenotype.

### 3.2 Accumulation of branched chain amino acids in plasma and urine of *Bcat2*^p.Q300*/p.Q300*^ mice

BCAT2 is located in the mitochondria of most cells of the body and is responsible for catabolizing BCAAs (leucine, isoleucine, and valine), essential amino acids that must be obtained from dietary sources. Following catabolism to their respective α-keto acids (BCKA), BCAAs are further catabolized by the branched-chain keto acid dehydrogenase complex (BCKDC), and the final catabolic products (acetyl-CoA and succinyl-CoA) are ultimately consumed in mitochondria for respiration through the tri-carboxylic acid cycle.^24^ In line with this, NMR analysis of plasma revealed an up to 10-fold increase in plasma levels of BCAAs (but no increase in BCKAs, which were under the detection threshold) in *Bcat2*^p.Q300*/p.Q300*^ mice aged 5 weeks (*Figure 2B*–*D*). Similar accumulation of BCAAs was observed in the urine of mutant mice aged 5 weeks (Supplementary material online, *Figure S4*). Hence, the identified mutation resulted in a truncated BCAT protein with consequent accumulation of BCAAs but not BCKAs. Heterozygous *Bcat2^+/^*^p.Q300*^ mice did not display sudden death or any other overt phenotype up to the age of 6–8 months, showed normal BCAA plasma levels (*Figure 2B*–*D*), and hence were not investigated further.

### 3.3 *Bcat2*^p.^^Q300*/p.Q300*^ mice display cardiac electrical alterations and increased arrhythmia inducibility

**Figure 3:**
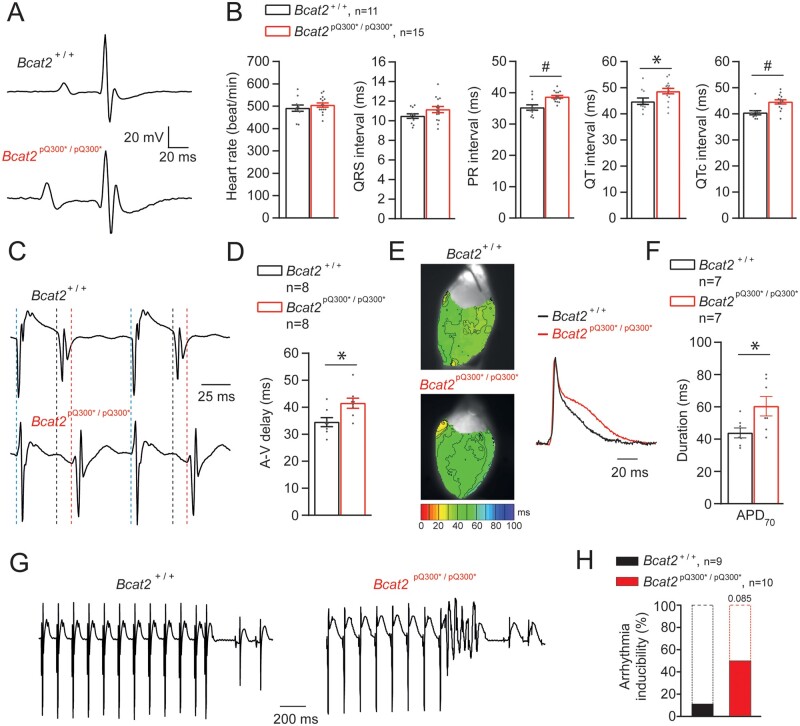
Homozygous *Bcat2*^p.Q300*/p.Q300*^ mice display ECG abnormalities *in vivo* and atrio-ventricular delay, prolonged repolarization and arrhythmia inducibility *ex vivo*. (*A*) Typical ECG traces obtained under isoflurane anaesthesia for *Bcat2*^+/+^ and *Bcat2*^p.Q300*/p.Q300*^ mice. (*B*) Average values for heart rate, QRS interval, PR interval, QT and QTc (corrected) for both *Bcat2*^+/+^ (*n* = 11) and *Bcat2*^p.Q300*/p.Q300*^ (*n* = 15) mice aged 4–5 weeks. (*C*) Typical examples of atrio-ventricular (AV) delay measurements in Langendorff-perfused isolated hearts (right atrial stimulation at a basic cycle length of 120 ms). (*D*) Average values for AV-delay in *Bcat2*^+/+^ and *Bcat2*^p.Q300*/p.Q300*^ hearts. (*E*) Typical left ventricular repolarization maps obtained from optical mapping experiments on perfused explanted hearts during central stimulation (6.6 Hz). (*F*) Average APD_70_ (action potential duration at 70% of repolarization) values for *Bcat2*^+/+^ (*n* = 7) and *Bcat2*^p.Q300*/p.Q300*^ (*n* = 7) mice. *P < 0.05, Student’s *t*-test. (*G*) Typical example of a short run of ventricular tachycardia induced in an isolated, Langendorff-perfused *Bcat2*^p.Q300*/p.Q300*^ heart following a short-coupled paced beat. (*H*) Bar graph representing the percentage of arrhythmic events recorded on *Bcat2*^+/+^ and *Bcat*^p.Q300*/p.Q300*^ mouse explanted hearts. *P < 0.05, Student’s *t*-test.

Given the absence of overt prior symptoms in *Bcat2*^p.Q300*/p.Q300*^ mice, we hypothesized a cardiac basis for their sudden death. No cardiac structural changes such as hypertrophy, dilation or fibrosis were observed in *Bcat2*^p.Q300*/p.Q300*^ hearts upon histological examination (Supplementary material online, *Figure S5A*–*C, Table S2*). We therefore performed detailed electrophysiological analysis in mice aged 4–5 weeks, well before the age of 6 weeks at which they started dying suddenly. Due to their young age and hence small size, and considering needed recovery time post-surgery, *in vivo* telemetric Holter monitoring was not possible. However, surface ECGs under isoflurane anaesthesia revealed significantly prolonged QT, QTc and PR intervals in *Bcat2*^p.Q300*/p.Q300*^ compared to *Bcat2*^+/+^ mice (*Figure 3A and B*, Supplementary material online, *Table S2*). We next investigated the *ex vivo* cardiac electrophysiological differences between *Bcat2*^+/+^ and *Bcat2*^p.Q300*/p.Q300*^ isolated, Langendorff-perfused hearts. Atrioventricular (AV) conduction time was significantly increased in *Bcat2*^p.Q300*/p.Q300*^ mice (*Figure 3C and D*, Supplementary material online, *Table S2*), similar to the increased PR-interval observed on ECG analysis. Optical mapping experiments revealed no differences in ventricular conduction between the groups (Supplementary material online, *Figure S6A* and *B, Table S2*). Nevertheless, repolarization time (APD_70_) was significantly prolonged in *Bcat2*^p.Q300*/p.Q300*^ compared to *Bcat2*^+/+^ hearts (60.4 ± 6.0 ms and 43.9 ± 3.1, respectively; *P* < 0.05—*Figure 3E and F*), in line with the observed QTc-prolongation on the surface ECG. Furthermore, the difference between the longest and shortest APD_70_ was also significantly increased in *Bcat2*^p.Q300*/p.Q300*^ (16.3 ± 1.3 ms) as compared to *Bcat2*^+/+^ hearts (9.1 ± 1.3 ms; *P* < 0.05) (Supplementary material online, *Table S2*), suggesting increased pro-arrhythmic APD heterogeneity. Arrhythmia inducibility studies in Langendorff-perfused hearts showed an increased incidence of inducible arrhythmias in *Bcat2*^p.Q300*/p.Q300*^ mice compared to *Bcat2^+/+^* (50% vs. 11.1%, respectively), the majority of which were non-sustained (*Figure 3G and H*).

### 3.4 Action potential prolongation, calcium dysregulation, and pro-arrhythmic events in *Bcat*^p.^^Q300*/p.Q300*^ cardiomyocytes

**Figure 4:**
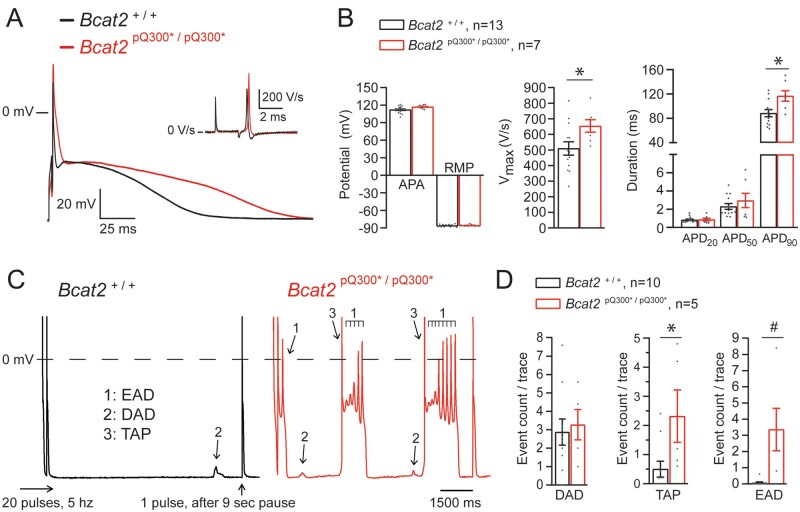
Abnormal repolarization and pro-arrhythmic events in *Bcat*^p.Q300*/p.Q300*^ isolated cardiomyocytes. (*A*) Typical examples of action potentials (AP) and upstroke velocities (dV/dt_max_; inset) elicited at a stimulation frequency of 2 Hz in left ventricular (LV) isolated cardiomyocytes from *Bcat2*^+/+^ and *Bcat2*^p.Q300*/p.Q300*^ mice aged 4–5 weeks. (*B*) Average values for APA (action potential amplitude), RMP (resting membrane potential), *V*_max_ (upstroke velocity), and APD (action potential duration) at 20%, 50%, and 90% repolarization (APD_20_, APD_50_, and APD_90_) of LV cardiomyocytes isolated from *Bcat2*^+/+^ (*n* = 15 cells from 6 independent cardiomyocyte dissociations) and *Bcat2*^p.Q300*/p.Q300*^ (*n* = 9 cells from 5 independent cardiomyocyte dissociations) mice. (*C*) Typical examples of early afterdepolarizations (EADs: 1), delayed afterdepolarizations (DADs: 2), and triggered action potentials (TAPs: 3) recorded after a fast pacing stimulation protocol (20 pulses at 5 Hz followed by a 9 s pause and 1 pulse followed by a 500 ms pause) in *Bcat2*^+/+^ and *Bcat2*^p.Q300*/p.Q300*^ LV cardiomyocytes. (*D*) Average count per trace for DADs, TAPs and EADs observed in *Bcat2*^+/+^ (*n* = 10 cardiomyocytes from 6 mice) and *Bcat2*^p.Q300*/p.Q300*^ mice (*n* = 5 cardiomyocytes from 4 mice). Average numbers were calculated using five consecutive traces. (with Student’s *t*-test or Mann–Whitney Rank sum test).

We next investigated the cellular electrophysiological alterations underlying the observed pro-arrhythmia in *Bcat2*^p.Q300*/p.Q300*^ mice. *Figure 4A* shows typical APs and maximal upstroke velocities (V_max_) elicited at a pacing frequency of 2 Hz in isolated left ventricular cardiomyocytes isolated from *Bcat2*^+/+^ and *Bcat2*^p.Q300*/p.Q300*^ hearts. V_max_, APA, and AP duration at 90% repolarization (APD_90_) were significantly increased in *Bcat2*^p.Q300*/p.Q300*^ compared to *Bcat2*^+/+^ cardiomyocytes (*Figure 4B*, Supplementary material online, *Table S4*). Using a fast pacing (20 pulses at 5 Hz) protocol, the presence of pro-arrhythmic events was evaluated, including EADs, DADs and TAPs (indicated by arrows in *Figure 4C*). The incidence of TAPs and EADs was significantly higher in *Bcat2*^p.Q300*/p.Q300*^ as compared to *Bcat2*^+/+^ cardiomyocytes (*Figure 4D*). The observed increase in V_max,_ APD_90_ and EAD incidence was not associated with alterations in sodium current: although *Bcat2*^p.Q300*/p.Q300*^ cardiomyocytes showed a hyperpolarizing shift in both activation and inactivation, the peak current density, window current, and late sodium current density were not altered (Supplementary material online, *Figure S7* and *Table S4*).

**Figure 5:**
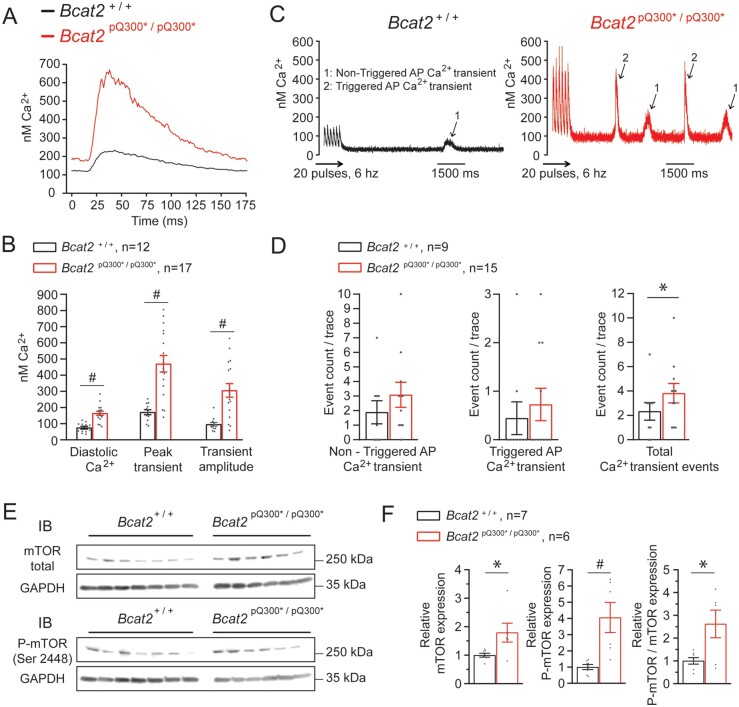
Abnormal intracellular calcium and myocardial mTOR upregulation in *Bcat2*^p.Q300*/p.Q300*^ mice. (*A*) Typical intracellular calcium transient recording performed at a pacing frequency of 6 Hz and (*B*) average values for diastolic Ca^2+^, peak transient and transient amplitude of LV cardiomyocytes from *Bcat2*^+/+^ (*n* = 12 cardiomyocytes from 5 independent cardiomyocyte dissociations) and *Bcat2*^p.Q300*/p.Q300*^ (*n* = 17 cardiomyocytes from 4 independent cardiomyocyte dissociations) mice aged 4-5 weeks. (*C*) Typical example of calcium after-transients in isolated cardiomyocytes from *Bcat2*^+/+^ and *Bcat2*^p.Q300*/p.Q300*^ cardiomyocytes following a fast pacing protocol using field stimulation and (*D*) quantification of non-triggered action potential (AP) Ca^2+^ transient, triggered AP Ca^2+^ transient and total Ca^2+^ transient events in *Bcat2*^+/+^ (*n* = 9 cardiomyocytes from 3 mice) and *Bcat2*^p.Q300*/p.Q300*^ mice (*n* = 15 cardiomyocytes from 4 mice). (*E*) Representative immunoblots of total mTOR, phosphorylated mTOR (P-mTOR) at ser2448 and GAPDH from heart lysates (apex) from *Bcat2*^+/+^ and *Bcat2*^p.Q300*/p.Q300*^ mice. (*F*) Average total mTOR and phosphorylated P-mTOR expression normalized to GAPDH, and ratio of normalized P-mTOR on normalized total mTOR from *Bcat2*^+/+^ (*n* = 7) and *Bcat2*^p.Q300*/p.Q300*^ (*n* = 6) mouse heart lysates.*P < 0.05; ^#^*P* ≤ 0.001 (with Student’s *t*-test or Mann–Whitney Rank sum test).

Since intracellular calcium dysregulation often underlies pro-arrhythmic DADs and TAPs, we next explored calcium homeostasis in isolated cardiomyocytes. *Figure 5A* shows typical calcium concentration ([Ca^2+^]_i_) transients elicited at a pacing frequency of 6 Hz. On average, *Bcat2*^p.^^Q300*/p.Q300*^ cardiomyocytes showed increased intracellular diastolic calcium levels ([Ca^2+^]_i_), higher [Ca^2+^]_i_ transients, and increased transient amplitudes as compared to *Bcat2*^+/+^ cells (*Figure 5B*, Supplementary material online, *Table S5*). SR content measured by rapid cooling was also significantly increased in *Bcat2*^p.Q300*/p.Q300*^ cardiomyocytes (Supplementary material online, *Figure S8A* and *B*), while the ratio between diastolic [Ca^2+^]_i_ and SR content was not different between groups (Supplementary material online, *Table S5*). Moreover, mRNA expression levels of *Slc8a1* [sodium/calcium exchanger (NCX)] and *Atp2a2* (SERCA) were unchanged (Supplementary material online, *Figure S9* and *Table S2*). *Figure 5C* shows typical examples of calcium after-transients recorded following a fast-pacing protocol (20 pulses at 6 Hz) using field stimulation. These calcium after-transients were separated into two groups based on their amplitude: (i) after-transients of low amplitude considered not to trigger an AP, and (ii) high-amplitude after-transients capable of triggering an AP (i.e. TAP). The total number of calcium after-transients (triggered and non-triggered combined) was significantly higher in *Bcat2*^p.Q300*/p.Q300*^ compared to *Bcat2*^+/+^ cardiomyocytes (*Figure 5C and D*, Supplementary material online, *Table S5*), indicating a calcium-dependent pro-arrhythmia mechanism. The addition of 50 nmol/L noradrenaline significantly increased diastolic [Ca^2+^]_i_, systolic [Ca^2+^]_i_ and transient amplitude in *Bcat2*^+/+^ cardiomyocytes, but only marginally in *Bcat2*^p.Q300*/p.Q300*^ cardiomyocytes (Supplementary material online, *Table S5*). Nevertheless, noradrenaline significantly increased the number of calcium after-transients in both *Bcat2*^+/+^ and *Bcat2*^p.Q300*/p.Q300*^ cardiomyocytes (Supplementary material online, *Table S5*).

### 3.5 Direct pro-arrhythmic effects of BCAAs in human PSC-derived cardiomyocytes

**Figure 6:**
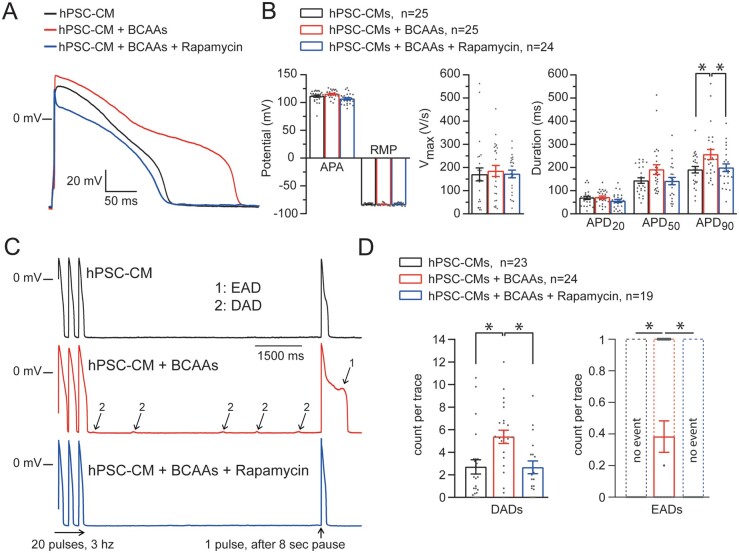
Increased levels of BCAAs recapitulate the pro-arrhythmic phenotype in hPSC derived cardiomyocytes (hPSC-CMs) which is reversible upon mTOR inhibition. (*A*) Typical example of APs elicited at a stimulation frequency of 1 Hz from hPSC-CMs incubated with control medium or with increased levels of BCAAs in the presence or absence of the mTOR inhibitor rapamycin (500 nmol/L). (*B*) Average values for APA (AP amplitude), RMP (resting membrane potential), *V*_max_ (upstroke velocity), and APD (action potential duration) at 20%, 50%, and 90% repolarization (APD_20_, APD_50_, and APD_90_) of hPSC-CMs incubated with the control medium (*n* = 25 hPSC-CMs from 5 dissociations), with increased levels of BCAAs (*n* = 25 hPSC-CMs from 5 dissociations), and with increased BCAA levels and rapamycin (*n* = 24 hPSC-CMs from 5 dissociations). (*C*) Typical examples of EADs (1) and DADs (2) recorded after a fast-pacing stimulation protocol (20 pulses at 3 Hz followed by an 8 s pause and 1 pulse followed by a 1 s pause). (*D*) Average count per trace for DADs and EADs observed in hPSC-CMs incubated with the control medium (*n* = 23 hPSC-CMs from 5 dissociations), with increased levels of BCAAs (*n* = 24 hPSC-CMs from 5 dissociations), and with increased BCAA levels and rapamycin (*n* = 19 hPSC-CMs from 5 dissociations). Average numbers were calculated using five consecutive traces. *P < 0.05, one-way ANOVA (with Student–Newman–Keuls Method *post hoc* test) or one-way ANOVA on ranks (with Dunn’s method *post hoc* test).

**Figure 7:**
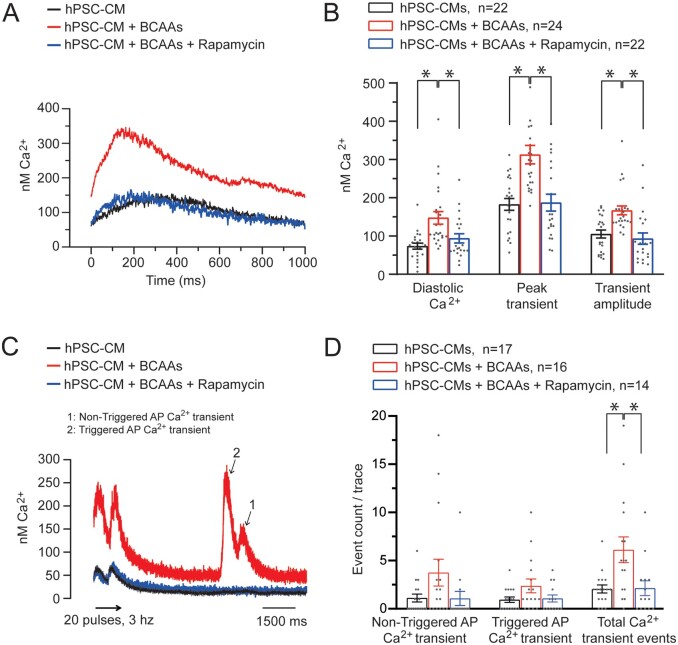
Abnormal intracellular calcium regulation in human PSC derived cardiomyocytes (hPSC-CMs) with elevated BCAAs is reversible upon mTOR inhibition. (*A*) Typical examples of intracellular calcium transient recordings in hPSC-CMs performed at a pacing frequency of 1 Hz. (*B*) Average values for diastolic Ca^2+^, peak transient and transient amplitude with control medium (*n* = 22 hPSC-CMs from 3 dissociations), increased BCAA levels (*n* = 24 hPSC-CMs from 3 dissociations), and with increased BCAA levels and rapamycin at 500 nmol.L^−1^ (*n* = 22 hPSC-CMs from 3 dissociations). (*C*) Typical example of calcium after-transients in hPSC-CMs incubated with a control medium or with increased levels of BCAAs following a fast-pacing protocol using field stimulation and (*D*) quantification of non-triggered action potential (AP) Ca^2+^ transient, triggered AP Ca^2+^ transient and total Ca^2+^ transient events in hPSC-CMs incubated with the control medium (*n* = 17 hPSC-CMs from 2 dissociations), with increased levels of BCAAs (*n* = 16 hPSC-CMs from 2 dissociations), and with increased BCAA levels and rapamycin (*n* = 14 hPSC-CMs from 2 dissociations). *P < 0.05, one-way ANOVA on ranks (with Dunn’s method *post hoc* test).

To demonstrate a direct causal link between elevated BCAAs and pro-arrhythmia, we evaluated the cellular electrophysiological consequences of increased BCAA levels in cultured hPSC-CMs. Incubation of hPSC-CMs for 5 days with BCAA levels similar to those observed in plasma of *Bcat2*^p.Q300*/p.Q300*^ mice resulted in significant prolongation of APD_90_ (*Figure 6A and B*, Supplementary material online, *Table S6*), and significantly increased the occurrence of pro-arrhythmic early and delayed afterdepolarizations (EAD/DAD) as compared to hPSC-CMs cultured in control medium (*Figure 6C and D*, Supplementary material online, *Table S6*), hence recapitulating the findings observed in *Bcat2*^p.Q300*/p.Q300*^ cardiomyocytes. Incubation of valine alone induced similar APD_90_ prolongation and increased EAD/DAD incidence, while alanine (as a general amino acid control) had no effects (Supplementary material online, *Figure S10A*–*D, Table S8*). Similar to *Bcat2*^p.Q300*/p.300*^ cardiomyocytes, hPSC-CMs cultured in medium with increased BCAA concentration also displayed intracellular calcium dysregulation, including increased diastolic [Ca^2+^]_i_ and after-transients (*Figure 7A*–*D*, Supplementary material online, *Table S6*), further confirming a direct pro-arrhythmic effect.

### 3.6 Pro-arrhythmic effects of elevated BCAA levels are mediated by mTOR pathway activation

In addition to their role in energy balance, BCAAs function as potent nutrient signal molecules to promote protein synthesis, cellular metabolism, and cell growth in a mammalian target of rapamycin (mTOR)-dependent manner,^24,25^ and elevated BCAA concentrations have been shown to lead to chronic induction of mTOR activity and increased oxidative stress.^26,27^ Indeed, cardiac tissue from *Bcat2*^p.Q300*/p.Q300*^ mice showed significantly higher expression levels of both mTOR total protein and activated P-mTOR as compared to *Bcat2*^+/+^ (normalized for GAPDH), in addition to an increased P-mTOR to mTOR ratio, indicating mTOR pathway activation (*Figure 5E and F*, Supplementary material online, *Table S2*). In line with mTOR pathway activation, the mRNA expression level of the pro-hypertrophic marker *Anf* was also significantly increased in *Bcat2*^p.Q300*/p.Q300*^ hearts (Supplementary material online, *Figure S9* and *Table S2*). The functional contribution of mTOR pathway activation to the observed pro-arrhythmic effects of BCAAs was subsequently demonstrated in hPSC-CMs. Incubation with the mTOR inhibitor rapamycin (500 nmol/L) prevented the APD_90_ prolongation and EAD/DAD incidence (*Figure 6A*–*D*), as well as the calcium dysregulation and after-transient occurrence (*Figure 7A*–*D*) induced by increased BCAA levels (Supplementary material online, *Table S6*), whereas it had no electrophysiological effects in the absence of high BCAA levels (Supplementary material online, *Table S7*). These observations demonstrate the functional involvement of mTOR pathway activation in the pro-arrhythmic effects of elevated BCAA levels.

### 3.7 Plasma BCAA levels correlate with ECG parameters in the general population

Finally, to confirm a modulatory role of BCAAs on cardiac electrical function in humans, we investigated the relation between ECG measures and BCAA plasma levels in 2304 participants (mean age 51.5 ± 10.6 years; 52.6% women) from the German community-based KORA F4 Study.^14^ Average ECG parameters were: heart rate 63.8 ± 9.7 b.p.m., PR interval 165.9 ± 23.2 ms, QRS duration 93.4 ± 11.8 ms, and QTc interval 422.3 ± 20.4 ms. The mean concentration of valine was 275.4 ± 60.2 µmol/L and of xleucine was 213.2 ± 45.2 µmol/L. Both valine and xLeucine followed a normal distribution, and showed a linear relation with ECG parameters (Supplementary material online, *Figure S11*). Regression results between ECG measures and BCAAs are presented in *Table 1*. We observed significant sex-adjusted associations between fasting valine plasma levels and the ECG parameters PR interval, QRS duration, and QTc interval. This association was consistent across men and women for the PR interval, and maintained effect directionality for QRS duration and QTc interval. Overall, the effect size was strongest for PR interval (beta: 0.0335 ± 0.0085 ms/µmol/L; *P* < 0.001), for which we also identified a significant association with xLeucine (i.e. the combination of Leucine and Isoleucine; beta: 0.0270 ± 0.0125 ms/µmol/L; *P* < 0.05). Together with the observations in *Bcat2*^p.Q300*/p.Q300*^ mice, these findings indicate that higher plasma BCAA levels are associated with altered cardiac conduction and repolarization.

**Table 1:** Association of ECG measures with branched-chain amino acids in the KORA F4 Study

		Unstratified		Men		Women	
ECG measure	BCAA	Beta (SE)	*P*-value	Beta (SE)	*P*-value	Beta (SE)	*P*-value
Heart rate (b.p.m.)	Valine (µmol/L)	0.0035 (0.0036)	0.34	0.0017 (0.0053)	0.75	0.0054 (0.0050)	0.28
	×Leucine (µmol/L)	0.0026 (0.0053)	0.61	0.0051 (0.0073)	0.49	−0.0004 (0.0077)	0.96
PR (ms)	Valine (µmol/L)	0.0335 (0.0085)	**<0.001**	0.0264 (0.0126)	**0.036**	0.0410 (0.0115)	**<0.001**
	×Leucine (µmol/L)	0.0270 (0.0125)	**0.031**	0.0226 (0.0175)	0.20	0.0327 (0.0179)	0.07
QRS (ms)	Valine (µmol/L)	−0.0086 (0.0042)	**0.040**	−0.0134 (0.0062)	**0.030**	−0.0034 (0.0056	0.54
	×Leucine (µmol/L)	−0.0069 (0.0061)	0.26	−0.0134 (0.0086)	0.12	0.0016 (0.0087)	0.86
QTc (ms)	Valine (µmol/L)	0.0234 (0.0075)	**0.002**	0.0173 (0.0108)	0.11	0.0297 (0.0104)	**0.004**
	×Leucine (µmol/L)	0.0144 (0.0110)	0.19	0.0032 (0.0150)	0.83	0.0287 (0.0161)	0.08

Linear regression models excluded participants ≤50 kg body weight, ≥70 years of age, or with diabetes mellitus. Unstratified models adjusted for sex.

Beta, standardized regression coefficient; *P, P*-value; SE, standard error. Data were considered significant below a P-value of 0.05 (indicated in boldface).

## 4. Discussion

Through a phenotype-driven random mutagenesis screen^11^ we here identified a mutant mouse line presenting with a striking, highly penetrant phenotype of sudden death without prior symptoms. The causative mutation was determined to be a nonsense mutation in the *Bcat2* gene resulting in a defect in BCAA catabolism and the accumulation of high levels of BCAAs in plasma and urine. Detailed electrophysiological studies unravelled a robust arrhythmogenic phenotype in affected mice, and additional studies in hPSC-CMs confirmed a direct pro-arrhythmic effect of elevated BCAAs levels. We therefore identified for the first time a role for BCAA dysregulation in modulating cardiac electrophysiology and risk for cardiac arrhythmias and sudden death.

BCAT2 is located in the mitochondria of most cells of the body, and is responsible for catabolizing branched chain amino acids (BCAAs; leucine, isoleucine, and valine), essential amino acids that must be obtained from dietary sources. Following catabolism by either BCAT2 or BCAT1 (the cytosolic isoform) to their respective α-keto acids (BCKA), BCAAs are further catabolized by the BCKDC.^24^ The final catabolic products of BCAAs are consumed in mitochondria for respiration through the tri-carboxylic acid cycle. Defects in BCAA catabolism have been associated with the development of Maple Syrup Urine disease (MSUD), an autosomal recessive metabolic disorder associated with brain damage caused by mutations in genes of the branched chain BCKDC complex.^24^ Here, the block in BCAA catabolism prevents the catabolism of BCKAs, the catabolic product resulting from the action of the two BCAT enzymes, cytosolic and mitochondrial. MSUD patients exhibit a failure to thrive and neurological deterioration thought to be associated with the accumulation of BCKAs.^24^ This was mirrored in a different, previously published mouse line with an ENU-induced *Bcat2*, which resulted in the 5′ splice site in *Bcat2* and the deletion of exon 2 with an absence of mature BCAT2 protein.^28^ These mutant mice displayed failure to thrive, reduced body weight and early death; no details were provided on the early death phenotype, and cardiac (electrophysiological) characterization was not performed. Analysis of the plasma revealed increased levels of both BCAAs (albeit it to a lower extent as compared to *Bcat2*^p.Q300*/p.Q300*^ mice) and BCKAs, with the latter prevented from entering the mitochondria and therefore accumulating. In contrast, our *Bcat2*^p.Q300*/p.Q300*^ mutation results in the production of a truncated protein, no differences in body weight, an accumulation of BCAAs but not BCKAs, and a sudden death phenotype. In particular, the absence of neuronal damage is likely due to the fact that BCKAs in *Bcat2*^p.Q300*/p.Q300*^ mice were not elevated and hence glutamate signalling not disturbed. Mice with targeted deletion of exon 4–6 of *Bcat2* demonstrated lower body weight and exercise intolerance, while their BCKA plasma levels remained normal.^29^ The lack of accumulation of BCKAs in our mice implies that the BCKA transport mechanism is intact in *Bcat2*^p.Q300*/p.Q300*^ mice but not in the previously described *Bcat2* deficient mice, as catabolism of BCKAs occurs within the mitochondria.^24^ This suggests that the truncated BCAT2^Q300*^ protein has impaired transamination activity yet retained BCKA transport activity^30,31^ either in the truncated protein or by maintaining the interaction of BCAT2 with a separate transport protein. This is supported by the observation that in our mice the concentration of valine is higher than that observed in the null mice. Hence, our novel mouse model presenting specific BCAA accumulation in the absence of BCKAs allows the investigation of the physiological effects of an excess of BCAAs, and the dissection of the transamination and BCKA transport activities of BCAT2. Additionally, all murine *Bcat2* models described so far were generated in C57BL/6J mice, a murine strain known to present distinct mitochondrial calcium and reactive oxygen species (ROS) handling properties demonstrated to be protective in heart failure models.^32^ In our present study, after the initial ENU mutagenesis step, mice were backcrossed onto the C3H background strain which may explain the severe phenotype severity observed in *Bcat2*^p.Q300*/p.Q300*^ mice.

Our findings identify a profound effect of elevated BCAA levels on intracellular calcium handling. Despite the substantial increase in diastolic and systolic [Ca^2+^]_i_ in *Bcat2*^p.^^Q300*/p.Q300*^ cardiomyocytes, the Ca^2+^ gradient across the SR membrane and the decay time of the Ca^2+^ transient remained unaltered, arguing against underlying alterations in phosphorylation state. Taken together, we consider the altered diastolic [Ca^2+^]_i_ as the main driver for the observed pro-arrhythmic calcium dysregulation in *Bcat2*^p.^^Q300*/p.Q300*^ cardiomyocytes. Elevated diastolic Ca^2+^, which has been shown to increase both the systolic Ca^2+^ as well as the SR Ca^2+^ content,^33^ may be the consequence of various processes affecting ionic gradients across cell membranes, including altered energetic metabolism. Together with the increase in SR Ca^2+^, elevated diastolic Ca^2+^ leads to a higher open probability of the ryanodine channels and the likelihood of spontaneous Ca^2+^ release from the SR. Subsequent calcium removal by the NCX may then lead to membrane (after)depolarization and onset of arrhythmia. Of note, late sodium current density was not altered and hence unlikely to have contributed to the AP prolongation, EAD incidence, and Ca^2+^ dysregulation observed in in *Bcat2*^p.^^Q300*/p.Q300*^ cardiomyocytes. In line with the *in vivo* and *ex vivo* findings in the *Bcat2*^p.Q300*/p.Q300*^ mice, AV conduction (PR interval), and repolarization (QT interval) ECG traits were significantly correlated with BCAA plasma concentrations in the KORA cohort. These results indicate a modulatory effect of BCAAs on electrophysiological characteristics of both the working myocardium and the cardiac conduction system, suggesting that ventricular arrhythmias as well as conduction defects could be triggered by elevated BCAA levels. Conduction through the central part of the AV-node is mainly calcium-driven, and we have recently demonstrated that intracellular calcium dysregulation may significantly contribute to AV-conduction.^34^ Hence, BCAA-induced calcium dysregulation may affect AV-conduction through prolonged repolarization and/or reduced gap junctional conductance within the central AV-node. Additionally, while myocardial fibrosis was not increased in *Bcat2*^p.Q300*/p.Q300*^ mice, minor structural remodelling within the AV-nodal area may still have impacted on AV-conduction. Finally, the AV-nodal region is highly innervated and a modulatory role of BCAAs on neuronal function has been previously demonstrated in neurons,^35^ providing another potential mechanism by which AV-conduction may have been affected. Interestingly, gender differences in electrophysiological effects of BCAAs appear to be present in both humans (*Table 1*) and mice (see Supplementary material online, *Table S9*), which may be due to intrinsic BCAA concentration differences between men and women,^36^ and/or gender-related differences in ion channels, exchangers and calcium handling.^37,38^

Importantly, the electrophysiological changes and pro-arrhythmic events observed in hPSC-CMs incubated with elevated BCAA levels confirm a direct pro-arrhythmic effect of BCAAs in cardiomyocytes. To date, *BCAT2* human mutations have been reported in only six individuals, associated mainly with developmental delay and autistic features.^39^ Affected individuals displayed elevated plasma BCAA levels of up to 4 mmol/L (valine), with plasma BCKA levels remaining unchanged as in *Bcat2*^p.Q300*/p.Q300*^ mice. Interestingly, valine levels were generally more elevated than leucine and isoleucine in *BCAT2* mutation carriers,^39,40^ similar to our findings in *Bcat2*^p.Q300*/p.Q300*^ mice. Valine appears to play a central role in BCAA-induced electrophysiological abnormalities, as evidenced by our findings in hPSC-CMs that increased levels of valine only were sufficient to induce pro-arrhythmic effects. Moreover, we observed the strongest correlation with ECG parameters for valine in the KORA cohort. While MSUD patients also have elevated BCAA plasma levels, arrhythmias have not been reported but cardiac electrophysiological assessment is likely not routinely performed. Similarly, arrhythmias have as yet not been reported in *BCAT2* mutation carriers, but our results suggest that an in-depth cardiac electrophysiological investigation and follow-up could be beneficial for these patients as well as individuals suffering MSUD.

Acute BCAA administration has been shown to reduce infarct size and exert cardioprotective effects during myocardial ischemia-reperfusion.^41^ In contrast, chronic BCAA administration was found to exacerbate cardiac dysfunction and remodelling following myocardial infarction, and furthermore increased myocardial vulnerability to ischaemia–reperfusion injury by enhancing glycolysis and fatty acid oxidation.^42,43^ Taken together, these findings and our current observations suggest time-dependent effects triggered by BCAAs, with acute administration presenting potential benefits while a chronic exposure would be deleterious for cardiac (electrical) function. Crucially, we here demonstrate pro-arrhythmic actions of chronically elevated BCAA levels through direct effects on the cardiomyocyte level outside of the cardiac ischaemia–reperfusion context.

The striking rapamycin-mediated reversal of pro-arrhythmic events in hPSC-CMs as well as the absence of effect of rapamycin in non-BCAA treated cells suggest a central role of mTOR on the electrophysiological dysfunctions triggered by elevated BCAAs. A number of studies have implicated increased ROS production secondary to mTOR pathway activation in the detrimental effects of BCAAs supporting our current observations.^35,44^ ROS-driven oxidative stress is known to induce pro-arrhythmic calcium dysregulation in cardiomyocytes as observed in our *Bcat2*^p.Q300*/p.Q300*^ mice.^45^ Interestingly, two recent studies have shown that low-BCAA diets can improve life-span through a decreased activation of the mTOR pathway, suggesting that dietary intervention could represent a preventive option in patients with elevated BCAA plasma levels.^46,47^ The activation of the mTOR molecular pathway has also been linked to cardiac hypertrophy and structural remodelling.^48,49^ While *Bcat2*^p.Q300*/p.Q300*^ mice showed absence of fibrosis and normal cardiac dimensions, they did display increased ventricular transcript levels of *Anf*, indicating that pro-hypertrophic remodelling, likely secondary to mTOR pathway activation, preceded sudden death. We hypothesize that BCAA levels in affected mice rose drastically within a short period of time following weaning, causing severe electrophysiological changes and sudden death before more overt cardiac remodelling such as fibrosis could occur. BCAAs have also been shown to regulate AMP-activated protein kinase (AMPK),^25^ which functions as a metabolic sensor in cardiomyocytes. Interestingly, AMPK can directly modulate sarcolemmal ion channels and transporters, providing an additional pathway through which BCAAs can impact on cardiomyocyte electrophysiology.^50,51^ Crucially, the pro-arrhythmic potential of the clinically observed increased BCAA levels may be enhanced by conditions that further compromise either BCAA catabolism or cardiac conduction and repolarization, such as for instance metabolic disturbances, certain QT-prolonging drugs, and structural alterations (cardiac hypertrophy, fibrosis). BCAA dysregulation is furthermore an increasingly recognized feature of diabetes mellitus, obesity, and heart failure, acquired disorders associated with an increased risk for arrhythmias and SCD.^4–6,15–17,52^ Our current observations suggest that enhanced BCAA plasma levels as observed in metabolic syndromes may underlie, at least in part, the increased susceptibility to cardiac arrhythmias and SCD observed in these patients, constituting a potential target for the prevention and treatment of cardiac arrhythmias in this setting.

### 4.1 Limitations

The ENU mutagenesis approach is a powerful tool for identifying potential novel genes and pathways but is inherently limited by the fact that a potential role for additional ENU-induced mutations besides *Bcat2*^p.Q300*/p.Q300*^ cannot be completely ruled out. Nevertheless, the sudden death phenotype remained after backcrossing the mice onto another genetic background, strengthening the involvement of the *Bcat2*^p.Q300*/p.Q300*^ mutation. Importantly, the identification of the *Bcat2* mutation led to the discovery of elevated BCAA plasma levels in affected mice and the subsequent establishment of their pro-arrhythmic effects in an independent system (hPSC-CMs). While we were not able to establish whether *Bcat2*^p.Q300*/p.Q300*^ mice died from cardiac arrhythmia (given the young age at which sudden death occurred), the robust arrhythmogenic electrophysiological alterations in both *Bcat2*^p.Q300*/p.Q300*^ mouse cardiomyocytes and hPSC-CMs incubated with elevated levels of BCAAs confirmed a pro-arrhythmic effect of the latter. *Bcat2*^p.Q300*/p.Q300*^ mice presented with severely elevated BCAA levels whereas BCAA plasma levels in the KORA cohort were markedly lower. However, KORA participants provided a fasting blood sample, were not supplementing BCAAs for nutritional purposes, and were considered healthy (i.e. not metabolically compromised). Hence, even under normal circumstances in the general population, BCAAs are associated with cardiac conduction and repolarization. It will be an interesting future investigation to study if strongly increased BCAA levels in humans, as it is the case with nutritional supplementation or in the setting of metabolic disorders such as obesity or diabetes, result in even more pronounced ECG changes and/or arrhythmias. Milder elevations of BCAA levels following a longer exposure may also trigger cardiac structural remodelling through mTOR pathway activation. Both electrophysiological and structural consequences may be of significance in patients presenting an altered BCAA metabolism as well as in athletes supplementing their diet with BCAAs.

## 5. Conclusion

Using an unbiased approach, we have identified BCAA metabolism as a novel modulator of cardiac electrical function and a mediator of cardiac arrhythmias and sudden death. This new mechanism linking BCAAs to pro-arrhythmia may not only be relevant for inherited BCAT2 deficiency, but also for acquired metabolic disorders such as diabetes, obesity, and heart failure in which BCAA metabolism is impaired. Since cellular BCAA metabolism is reflected by BCAA plasma levels, the latter may serve as a circulating prognostic biomarker of arrhythmia and SCD risk. Moreover, such metabolic mechanisms are modifiable through e.g. dietary interventions, providing novel preventive and therapeutic targets that overcome the drawbacks of conventional anti-arrhythmic approaches targeting ion channels or transporters. Additionally, we here provide the first proof of concept that pharmacological inhibition of the mTOR pathway can abolish the pro-arrhythmic events provoked by elevated BCAAs. Overall, our findings pave the way for novel research into therapeutic strategies aimed at preventing electrical dysfunction and arrhythmogenesis in patients suffering from diabetes, obesity, and heart failure.

## Supplementary material

Supplementary material is available at *Cardiovascular Research* online.

## Authors’ contributions

V.P., C.R.B., P.K.P., and C.A.R. conceived and designed the project. P.K.P. lead the early description of the sudden death phenotype and identification of mutants. T.N. performed the initial sudden death phenotypic characterization and mutant characterization. S.F. and A.B. carried out phenotypic analysis. I.J.C., F.P., and T.A.H. acquired and analysed urinary and plasma NMR data. V.P., S.P., G.A.M., A.B., S.C., and A.O.V. performed electrophysiological measurements and data analysis. M.F.S., M.W.T.T., V.P., R.T., J.L.T., M.M.N., G.K., C.G., A.P., and S.K. participated in the KORA data analysis. V.P., T.N., and L.B. performed western blots and qPCR experiments. C.R.B., P.K.P., and C.A.R. supervised the project. V.P., C.R.B., P.K.P., and C.A.R. wrote the manuscript. All co-authors critically revised the manuscript for intellectual content.

**Conflict of interest:** None declared.

## Funding

This work was supported by grants from the Dutch Heart Foundation (CVON-PREDICT2 and CVON-eDETECT; to C.R.B. and C.A.R.), the Netherlands Organization for Scientific Research (Off Road fellowship, 451001031, to V.P.; VICI fellowship, 016.150.610, to C.R.B.; VIDI fellowship, 91714371, to C.A.R.), the Philippa and Marvin Carsley Chair in cardiology (R.T.), a Fondation Leducq Transatlantic Network of Excellence (C.R.B. and C.A.R.) and by the Medical Research Council, UK (MCU142684172 and MC_PC_13045, P.K.P.). R.T. is a clinical research scholar of Fonds de recherche du Québec—Santé. J.L.T. is supported by a 2019 NARSAD Young Investigator Grant from the Brain & Behavior Research Foundation.

## Data availability

The data underlying this article will be shared on reasonable request to the corresponding authors.

**Translational perspectives**

Development of efficient anti-arrhythmic strategies is hampered by incomplete knowledge of disease mechanisms. Using an unbiased approach, we here identified for the first time a pro-arrhythmic effect of increased levels of branched chain amino acids (BCAAs). This is of particular relevance for conditions associated with BCAA dysregulation and increased arrhythmia risk, including heart failure, obesity and diabetes, as well as for athletes supplementing their diet with BCAAs. Such metabolic dysregulation is potentially modifiable through dietary interventions, paving the way for novel preventive strategies. Our findings furthermore, identify mTOR inhibition as a potential anti-arrhythmic strategy in patients with metabolic syndrome.

## Supplementary Material

cvab207_Supplementary_Data

## Notes

Time for primary review: 24 days
