# Supplementary material for: Chronically elevated branched chain amino acid levels are pro-arrhythmic
Source: Cardiovasc Res. 2021 Jun 17;118(7):1742–57. doi: 10.1093/cvr/cvab207 (PMC9215196; doi:10.1093/cvr/cvab207)
Supplement: cvab207_Supplementary_Data [file cvab207_supplementary_data.docx]

**SUPPLEMENTAL MATERIAL**

***Chronically elevated branched chain amino acid levels are pro-arrhythmic***

***Portero et al.***

**Expanded Materials & Methods**

**Pyrosequencing**

DNA was extracted from mouse ear biopsies using Kapa express extract (Sigma Aldrich). PCR was performed (Taq PCR master mix – Qiagen) focused on a region surrounding each mutation with biotinylated reverse primers (*Bcat2* F – GTAACCCCCCCACTGAAT, *Bcat2* R – AACTCACCCCAAGTCCTG, *Siglech* F – ACGACCACACTCTCCTCAG, *Siglech* R – AGGTGACATTGAGCTGGATAG). Following PCR, the biotinylated strand was isolated by denaturation and washing, and pyrosequencing performed (PSQ HS96A pyrosequencer, Pyromark reagents). Sequencing primers are added (*Bcat2* – TGCCTGGAGTAGTTCG, *Siglech* - AACCAACCTCACCTGT) as well as enzyme and substrate (Qiagen). Successive addition of nucleotides provides a luminescent signal incorporated into the DNA strand through synthesis. Pyrosequencing results are presented in **Suppl. Fig. S1**.

**NMR methodology**

***Urinary NMR analysis***

Free-fed mouse urine samples were collected by metabolic caging overnight and stored at -80 ^o^C the following day until NMR analysis. Urinary samples were collected from two batches of mice. Firstly, batch A from the initial ENU screen and secondly, batch B from the *Bcat2* backcrossed congenic line. From batch A, 18 urine samples were collected (*Bcat2*^+/+^, n=10, mean (range) age 4.31 (4.29-4.57) weeks; *Bcat2*^p.Q300*/p.Q300*^, n=8, mean (range) age 4.10 (4.00-4.29) weeks) and analysed using a JEOL Eclipse+ 500MHz NMR system housed at MRC Harwell. From batch B, 10 urine samples from the congenic line mice were collected (*Bcat2*^+/+^, n=5, mean (range) age 4.03 (3.86-4.14) weeks; *Bcat2*^p.Q300*/p.Q300*^, n=5, mean (range) age 4.14 (4.14) weeks) and subsequently analysed using a Bruker AV 700 MHz NMR system at the Department of Chemistry, University of Oxford. Both female and male mice were included in the NMR urinary BCAA quantification.

In preparation for NMR analyses, the urine samples were briefly thawed at room temperature and centrifuged at 8,000 rpm for 2 minutes. A urine volume was mixed with sterile water and 0.2M phosphate buffer (pH7.4, containing 20% D_2_O to provide an NMR field frequency lock and 1 mM trimethylsilyl-propionic acid (TSP) for internal chemical shift reference at 0.00 ppm) in the proportions 1:2:3. At 500 Mz and 700 MHz, 100 µl was diluted and buffered to a volume of 600 µl, and 550 µl of this buffered urine was analysed in a 5 mm NMR tube. Water pre-saturation was used for all data acquisitions. The spectral width was 15ppm, pulse angle 90^o^, acquisition time 4.36s, relaxation delay 2s and constant receiver gain. The free induction decay was zero filled and multiplied by an exponential function of 0.3Hz line broadening prior to Fourier Transformation. The NMR spectra were manually phased using proprietary software. NMR spectral resonances were assigned according to the literature.^1^ The BCAA region was integrated relative to the total signal intensity in the region (3.50-1.07 ppm).

***NMR plasma sample preparation and analysis***

Plasma samples were collected from 27 free-fed *Bcat2* backcrossed congenic line (*Bcat2*^+/+^, n=11, mean (range) age 4.83 (4.71-5.00) weeks; *Bcat2*^+/p.Q300*^, n=9, mean (range) age 4.90 (4.71-5.00) weeks; *Bcat2*^p.Q300*/p.Q300*^, n=7, mean (range) age 4.88 (4.71-5.00) weeks). All plasma samples were stored at -80^o^C until NMR analysis. Both female and male mice were included in the NMR plasmatic BCAA quantification.

In preparation for proton (^1^H)-NMR spectroscopy studies, plasma aliquots were thawed at 4 °C for 20 min and centrifuged for 5 min at 520 x g. A plasma volume of 100 µl was mixed with 100 µl D_2_O, which provided an NMR field frequency lock and the plasma solution was transferred to a 3 mm OD NMR tube (Norell®, USA). All plasma (^1^H)-NMR spectra were collected at 37°C using a Bruker AV 700 MHz NMR spectrometer housed in the Department of Chemistry, University of Oxford. NMR data were obtained using pulse-collect and spin-echo sequences. (^1^H)-NMR spectra were acquired using both noesygppr1d pulse-collect and Carr-Purcell-Meiboom-Gill (CPMG) spin-echo sequences, each with 32 data collects, acquisition time 1.46 s, relaxation delay 2 s and a fixed receiver gain. The resulting free induction decay was zero filled by a factor of 2 and multiplied by an exponential function corresponding to 0.3 Hz line broadening prior to Fourier Transformation. The (^1^H)-NMR spectra were manually phased using the Bruker TopSpin software (version 3.5 pl 5, Bruker Biospin Corporation, Billerica MA 01821) and referenced with the lactate doublet set to 1.33 ppm. (^1^H)-NMR spectral resonances were assigned according to the literature.^2^ Peaks in the BCAA region, 1.06 - 0.92 ppm, were assigned to valine, isoleucine and leucine (domain 10). The entire NMR spectrum was binned into regions of 0.02 ppm and integrated by the ACD/NMR Processor Academic Edition (version 12.01, Advanced Chemistry Development, Inc.)

***Estimate of relative contribution of plasmatic valine, isoleucine and leucine in a representative pulse-collect NMR spectrum at 700 MHz from Bcat2^p.Q300*/p.Q300*^***

Peaks were initially manually integrated in more detail from one representative plasma pulse-collect NMR spectrum from a *Bcat2*^p.Q300*/p.Q300*^ mouse using the Bruker TopSpin software integration routine. Appreciating the problems of peak overlap not only within the BCAA region, but also of overlap of the BCAA region with the lipid-CH_3_ peak and with any broad underlying lipoprotein resonances, in the NMR spectrum selected, the estimate of valine:isoleucine:leucine peak integrals was 1:0.75:0.45.

***Estimate of plasmatic valine concentration from CPMG spin-echo NMR spectra***

Using NMR data collected using the CPMG spin-echo sequence (to minimise overlap from lipid and lipoprotein signals) the NMR doublet at 1.06-1.02 ppm was assigned to a CH_3_ doublet of valine. The peak area of this region was compared to the signal level in the region 5.24-5.22 ppm, which was assigned to the alpha-anomeric proton of glucose. The valine signal was divided by 3, to correct for the number of protons contributing to this doublet. Assuming α-β anomeric equilibrium, the area integration of the alpha-anomeric proton was divided by 0.36.^3^ Similar spin-spin relaxation NMR values for the valine CH_3_ doublet and the anomeric glucose peak were assumed. Finally, assuming a glucose concentration of 17 mM, on the basis of archived clinical chemistry data for *Bcat2*^p.Q300*/p.Q300*^ mice, the concentration of valine was calculated from the valine CH_3_ signal area relative to the glucose α-CH signal area using a correction factor of (0.36/3) *17.0 = 2.04.

***Estimate of plasmatic xleucine concentration***

Since the NMR resonances from leucine and isoleucine (xleucine) overlap in the region 0.97-0.92 ppm,^4^ it is difficult to separate the contribution of these metabolites without the use of two-dimensional NMR techniques. Given that only one-dimensional NMR data were acquired, the xleucine concentration was estimated from the integral of the region 0.98-0.92ppm, divided by 4.5 (averaging the contribution of three protons to the isoleucine peak and six protons for the leucine peaks) and compared to the glucose anomeric doublet.

**Biochemical screen**

Further analysis of plasma was carried out by the clinical chemistry analysis of terminal plasma samples to determine if there were any other potential indicators of ill health; parameters measured are listed in **Suppl. Table S1**. Both female and male mice were included in the chemical chemistry screen. Clinical chemistry analysis was carried out on a Beckman Coulter AU680 analyser on terminal blood samples, acquired via retro-orbital bleeds into lithium heparin tubes under terminal anaesthesia. Blood samples were centrifuged at 5,000 x g for 10 minutes at 8°C and the resulting plasma was stored at -20^o^C prior to analysis. All analysis was carried out blinded using coded tubes.

**Cardiac structural abnormalities**

To assess the presence of cardiac hypertrophy, heart weight/body weight and heart weight/tibia length ratios were calculated. Histological assessment of cardiac tissue was carried out on haematoxylin and eosin (H&E) stained tissue obtained from 6-week-old G3 mice. Both female and male mice were included in the quantification.

**mRNA expression analysis**

Total RNA was extracted from ventricular tissue with Trizol (Sigma) following manufacturer’s recommendations and stored at -80^o^C. 1000 ng of isolated RNA was reverse-transcribed into cDNA with Superscript II (Invitrogen) and Oligo(dT) primers. mRNA expression of *Anf, Slc8a1, Atp2a2 and Hprt* (primer sequences: *Hprt*-forward: 5′_CTTTCCCTGGTTAAGCAGTACAG_3′, *Hprt*-reverse: 5′_GTCAAGGGCATATCCAACAACAAAC_3′; *Atp2a2*-forward: 5′_TCCATCTGCTTGTCCATGTCAC_3′, *Atp2a2*-reverse: 5′_GGAGCAGGAAGATTTGGTGGC_3′, *Slc8a1*-forward: 5′_CCAACAGCTGGAGAGAGCAG_3′, *Slc8a1*-reverse: 5′_GTAATCAAAACAGGAGGGCAGC_3′; *Anf*-forward: 5’_TTCCTCGTCTTGGCCTTTTG_3’, *Anf*-reverse: 5’_CCTCATCTTCTACCGGCATC_3’) was determined by RT-qPCR using SYBR Green (Roche) on the LightCycler 480 PCR system (Roche). Samples were first denatured at 95°C (5 minutes), followed by 45 cycles with denaturation at 95°C (10 seconds), annealing at 60°C (20 seconds), and extension at 72°C (20 seconds), followed by with a standard melting curve protocol. Melting curve analysis (LightCycler480, Roche) and size fractionation by agarose gel electrophoresis were used to confirm amplification of the expected product. The *Anf, Slc8a1, Atp2a2 and Hprt* transcript levels for each sample were analysed in triplicate using LinRegPCR.^5,6^ *Hprt* served as a reference gene for normalization*.*

**Surface ECG measurements**

Mice were anesthetized by isoflurane inhalation (4% for induction, 0.8-1.5 % volume in oxygen for maintenance). Surface ECG recordings were performed for 5 min in mice in the prone position with limb 23-gauge needle using the Powerlab acquisition system (ADInstruments). ECG analysis was done with LabChart7Pro software (ADInstruments). The signal average ECG (SAECG) was calculated for leads I and II and manually analysed for ECG indices (P-, PR-, QRS-, and QT-duration and heart rate, HR). The following formula was used to correct QT-intervals for RR-interval: QTc =QT/(RR/100)^1/2^. Finally, the averages of obtained SAECG parameters from lead I were calculated. Both female and male mice were included.

**Cardiac arrhythmia inducibility**

Mice were sacrificed by cervical dislocation after CO_2_ exposure. The excised heart was cannulated, placed on a Langendorff perfusion set-up, and perfused at 37°C with Tyrode’s solution (128 mmol/l NaCl, 4.7 mmol/l KCl, 1.45 mmol/l CaCl_2_, 0.6 mmol/l MgCl_2_, 27 mmol/l NaHCO_3_, 0.4 mmol/l NaH2PO_4_, and 11 mmol/l glucose [pH maintained at 7.4 by equilibration with a mixture of 95% O_2_ and 5% CO_2_]). Atria and ventricles were stimulated at a basic cycle length (BCL) of 120 ms (2 ms pulse duration, twice diastolic threshold). Inducibility of arrhythmias was evaluated using up to 3 extrastimuli (S1-S2-S3) (after 16 stimuli at BCL 120 ms), with progressively shortened coupling intervals of 10 ms until the capture was lost. Moreover, burst pacing was applied using 36 stimuli at BCL, followed by 40 shortly coupled stimuli at progressively shortened coupling intervals. Both female and male mice were included.

**Optical mapping measurements**

Optical mapping was carried out as previously described.^7^ Following excision and cannulation as described above, hearts were incubated in 10 ml Tyrode’s solution containing 15 μM Di-4 ANEPPS and mounted on an optical mapping setup and perfused at 37°C with Tyrode’s solution. To prevent movement artifacts, blebbistatin was added to Tyrode’s solution. Excitation light was generated by a 5-watt power LED lamp (filtered 510 ± 20 nm). Fluorescence (filtered >610 nm) was transmitted through a tandem lens system on CMOS sensor (100 × 100 elements; MICAM Ultima). Pacing was performed at a BCL of 120 ms at twice the diastolic stimulation threshold from the center of the ventricular epicardial surface. Optical action potentials were analyzed, and local activation defined as the maximum positive slope of the action potential was calculated using custom software. Measured local activation times were used to construct ventricular activation maps. To calculate conduction velocity (CV) in longitudinal and transversal directions, the difference in activation time was determined between at least three consecutive electrode terminals parallel (longitudinal) or perpendicular (transversal) to the direction of propagation, as seen in isochronal maps. Three calculated parallel values of CV in each of the two directions were averaged. Both female and male mice were included.

**Cardiomyocyte isolation**

Mouse hearts were excised, cannulated, and mounted on a Langendorff set-up (see above), and perfused at 37 °C for 8 min with normal Tyrode’s solution containing (in mmol/l): 140 NaCl, 5.4 KCl, 1.8 CaCl_2_, 1.0 MgCl_2_, 5.5 glucose, 5 HEPES; pH 7.4 (NaOH). Next, the heart was perfused for 8 minutes with a similar solution in which the calcium concentration was lowered to 1.08 × 10^−5^ mol/L (low calcium solution), and the enzymes Liberase Blendzyme type 4 (Roche Diagnostics, GmbH, Mannheim, Germany) and Elastase from porcine pancreas (SERVA Electrophoresis GmbH, Heidelberg, Germany) were added at a concentration of 0.055 and 0.008 mg/mL, respectively. Then, digested tissue was gently triturated in the low-calcium solution and single cardiomyocytes were washed twice in the low-calcium solution supplemented with BSA (1 mg/ml), and twice in normal Tyrode’s solution at 37 °C. Cells were stored at room temperature and used within 4 h. Only male mice were included in the cellular electrophysiological studies.

**Action potential measurements**

Action potentials (APs) were measured at 36 °C using normal Tyrode’s solution and were elicited at 2 Hz. Pipettes were filled with (in mmol/l): 125 K-gluconate, 20 KCl, 5 NaCl, 0.22 amphotericin-B, 10 HEPES, pH 7.2 (KOH). APs were elicited at 2 Hz by 2-ms, ≈1.2× threshold current pulses through the patch pipette. In single hPSC-CMs, APs were measured with similar conditions as for mouse cardiomyocytes, with the exception that the amount of amphotericin was doubled. Typically, hPSC-CMs have a small or even complete lack of the inward rectifying potassium current (I_K1_). Consequently, hPSC-CMs have a depolarized resting membrane potential (RMP) and are frequently spontaneously active.^8^ To overcome these conditions, we injected an in silico I_K1_ with kinetics of Kir2.1 channels through dynamic clamp,^9^ as previously described and validated.^10,11^ Consequently, cells became quiescent with a RMP of around −82 mV and APs were elicited at 1 Hz by 3 ms, ≈1.2-times the threshold current pulses through the patch pipette. Signals were low-pass filtered with a cut-off frequency of 5 kHz and digitized at 40 kHz. We analysed RMP, AP amplitude (APA), maximal AP upstroke velocity (V_max_) and APD at 20, 50, and 90% repolarization (APD_20_, APD_50_, and APD_90_, respectively). Data from 10 consecutive APs were averaged and potentials were corrected for the calculated liquid junction potential (15 mV). The fast pacing protocol used to count and quantify early after depolarizations (EADs), delayed after depolarizations (DADs) and triggered action potentials (TAPs) consisted in applying 20 pulse at a frequency of 5 Hz followed by a 10 second pause. The results were normalized per trace using 5 consecutive traces.

**Sodium current measurements**

The sodium current (I_Na_) was measured using the ruptured patch-clamp technique. Glass pipettes were filled with a solution containing (in mmol/l): 3.0 NaCl, 133 CsCl, 2.0 MgCl_2_, 2.0 Na_2_ATP, 2.0 TEACl, 10.0 EGTA, 5.0 HEPES; pH 7.2 (CsOH). I_Na_ peak measurements were performed in a bath solution containing (in mmol/l): 7.0 NaCl, 133 CsCl, 1.8 CaCl_2_, 1.2 MgCl_2_, 11.0 glucose, HEPES 5.0, nifedipine 0.005; pH 7.4 (CsOH). For late I_Na_ measurements, we replaced 123 mmol/l CsCl by NaCl. Series resistance and cell membrane capacitance were compensated for 80-90%. Signals were filtered at 5 kHz and digitized at 40 kHz. Peak I_Na_ was measured at room temperature (21°C) from a holding potential of -120 mV following steps of 5 mV from -130 mV to +30 mV, with a cycle length of 5 s (**Suppl. Fig. S7C**). I_Na_ was defined as the difference between peak current and steady-state current. I_Na_ density was calculated by dividing current amplitude by cell membrane capacitance (C_m_). C_m_ was determined by dividing the decay time constant of the capacitive transient in response to 5 mV hyperpolarizing steps from −40 mV, by the series resistance (Rs). Steady-state activation and inactivation curves were fitted using the Boltzmann equation I/I_max_=A/{1.0+exp[(V_1/2_-V)/k]} to determine V_1/2_ (membrane potential for the half-maximal (in)activation) and the slope factor k. Late I_Na_ were measured at 36 °C as TTX (30 μM)-sensitive current using a descending ramp protocol (Supplemental Figure S7A).

**Western blot analysis of WT and p.Q300* BCAT2 protein expression**

Full length cDNA clone of BCAT2 (Dharmacon) was ligated into pCMV6-AN-Myc vector (Origene) using the restriction enzyme sites SgfI and AscI. The ligated vector was propagated in Xl-10 gold *E.coli* (Agilent) and extracted using plasmid midi kit (Qiagen). The C1121T mutation was introduced by Q5 site directed mutagenesis (NEB) using the following primers (5’>3’): GAGTAGTTCGATAAAGTCTGCTG and CAGGCAAGATGACGCCAT. Plasmid DNA was transfected using JetPRIME transfection reagent (Polyplus transfection) into HEK293T cells grown in DMEM (High glucose, Glutamax) supplemented with 10% FBS and penicillin/streptomycin according to manufacturer’s instructions. 48 hours following transfection cells were lysed in 200µl of 1x LDS sample buffer (invitrogen) containing protease inhibitors (Roche). 10µl of cell lysate was loaded into a 4-12% bis-tris gels (Invitrogen) and run in 1xMOPS buffer. Proteins were transferred to PVDF membrane (GE Healthcare) in 1x NuPAGE transfer buffer (Invitrogen), 20% methanol, and NuPAGE antioxidant (Invitrogen) at 30 volts for 60 minutes. The membrane was blocked in 5% milk in PBS containing 0.1% tween for 1 hour, and primary antibody (Mouse anti-Myc 1:1000 – Origene) incubated overnight in 5% milk PBS-tween at 4°C. Following overnight incubation, the membrane was washed in PBS-tween and incubated in secondary antibody (goat anti-mouse IgG IRdye800CW 1:15000 – Li-COR) for 1 hour at room temperature. Signal was visualised on Li-Cor ClX scanner.

**Association between plasma BCAA levels and ECG measures in the KORA F4 Study**

***KORA cohort description***

The community-based KORA Study (Cooperative Health Research in the Augsburg Region) is conducted in Augsburg, Southern Germany. The KORA F4 Study recruited 3080 participants between 2006 and 2008.^12^ All individuals received a detailed characterization of demographic information and comorbid conditions. Peripheral blood from fasting individuals was drawn for biomarker analyses. In addition, all participants obtained a standard 12-lead electrocardiogram (ECG) after 10 minutes rest in supine position using the Hannover ECG System (HES, Hannover, Germany). For the current analysis, we used heart rate, PR interval, QRS duration, and the QTc interval, corrected using Bazett’s formula. All study participants provided written informed consent; the study was approved by the Bayerische Landesärztekammer.

***Branched-chain amino acid assessment***

All participants underwent metabolic profiling using the Biocrates AbsoluteIDQp150 kit (Biocates Life Sciences AG, Innsbruck, Austria), adhering to manufacturer recommendations as previously described.^12^ For the current analysis, we extracted µmol/L concentrations of the BCAAs valine and xleucine (i.e. the combination of leucine and isoleucine).

***KORA statistical analysis***

Of 3080 KORA F4 participants, 75 were excluded due to unavailability or non-interpretability of ECGs, or absence of sinus rhythm. An additional 109 individuals were excluded following quality control of metabolite measurements, leaving 2896 samples for analysis. To account for the known interaction of BCAA plasma concentrations with body weight and age, we restricted our cohort to participants ≥50 kg and ≤70 years of age. Given the interference of the anti-diabetic drug metformin with BCAA concentrations, we further excluded participants with diabetes mellitus. In summary, additional 592 participants were excluded, leaving 2304 individuals in the final data set. To determine a relation between ECG measures and BCAAs, we fitted linear regression models adjusting for sex. To rule out interactions with age or sex, we tested for statistical interaction by including multiplicative interaction terms. As we found no evidence of interaction, interaction terms were removed from the final models. All statistical analyses in KORA were performed using STATA 12.0 (StataCorp LP, College Station, TX, USA).

**Statistical analysis**

No statistical methods were used to predetermine sample size. Differences between two unpaired groups were assessed using two-tailed *t*-tests when following a normal distribution and Mann-Whitney Rank sum test if normality test failed. All statistical tests were performed using sigma stat 3.5 software (Systat Software, Inc). Variability in all plots and graphs is presented as the s.e.m. All *P* < 0.05 were considered to be significant. **P* < 0.05; ***P* ≤ 0.01; #*P* ≤ 0.001. Summary statistics are depicted in **Suppl. Tables S2-5**.

**References**

1. Wishart DS, Tzur D, Knox C, Eisner R, Guo AC, Young N, Cheng D, Jewell K, Arndt D, Sawhney S, et al. HMDB: the Human Metabolome Database. *Nucleic Acids Res*. 2007;35:D521-526.

2. Psychogios N, Hau DD, Peng J, Guo AC, Mandal R, Bouatra S, Sinelnikov I, Krishnamurthy R, Eisner R, Gautam B, et al. The human serum metabolome. *PLoS ONE*. 2011;6:e16957.

3. Ando I, Hirose T, Nemoto T, Totsune K, Imai Y, Takeuchi K, Fujiwara M. Quantification of molecules in (1)H-NMR metabolomics with formate as a concentration standard. *J Toxicol Sci*. 2010;35:253–256.

4. Ghosh S, Sengupta A, Chandra K. Quantitative metabolic profiling of NMR spectral signatures of branched chain amino acids in blood serum. *Amino Acids*. 2015;47:2229–2236.

5. Ramakers C, Ruijter JM, Lekanne Deprez RH, Moorman AFM. Assumption-free analysis of quantitative real-time polymerase chain reaction (PCR) data. *Neurosci Lett*. 2003;339:62–66.

6. Ruijter JM, Ramakers C, Hoogaars WMH, Karlen Y, Bakker O, van den hoff MJB, Moorman AFM. Amplification efficiency: Linking baseline and bias in the analysis of quantitative PCR data. *Nucleic Acids Res*. 2009;37.

7. Rivaud MR, Jansen JA, Postema PG, Nannenberg EA, Mizusawa Y, van der Nagel R, Wolswinkel R, van der Made I, Marchal GA, Rajamani S, et al. A common co-morbidity modulates disease expression and treatment efficacy in inherited cardiac sodium channelopathy. *Eur Heart J*. 2018;39:2898–2907.

8. Casini S, Verkerk AO, Remme CA. Human iPSC-derived cardiomyocytes for investigation of disease mechanisms and therapeutic strategies in inherited arrhythmia syndromes: strengths and limitations. *Cardiovasc Drugs Ther*. 2017;31:325–344.

9. Veerman CC, Kosmidis G, Mummery CL, Casini S, Verkerk AO, Bellin M. Immaturity of human stem-cell-derived cardiomyocytes in culture: fatal flaw or soluble Problem? *Stem Cells Dev*. 2015;24:1035–1052.

10. Portero V, Casini S, Hoekstra M, Verkerk AO, Mengarelli I, Belardinelli L, Rajamani S, Wilde AAM, Bezzina CR, Veldkamp MW, Remme CA. Anti-arrhythmic potential of the late sodium current inhibitor GS-458967 in murine *Scn5a*-1798insD^+/-^ and human *SCN5A*-1795insD^+/-^ iPSC-derived cardiomyocytes. *Cardiovasc Res*. 2017;113:829–838.

11. Meijer van Putten RME, Mengarelli I, Guan K, Zegers JG, van Ginneken ACG, Verkerk AO, Wilders R. Ion channelopathies in human induced pluripotent stem cell derived cardiomyocytes: a dynamic clamp study with virtual I_K1_. *Front Physiol*. 2015;6:7.

12. Riedl A, Wawro N, Gieger C, Meisinger C, Peters A, Roden M, Kronenberg F, Herder C, Rathmann W, Völzke H, et al. Identification of Comprehensive Metabotypes Associated with Cardiometabolic Diseases in the Population-Based KORA Study. *Mol Nutr Food Res*. 2018;62:e1800117.

**
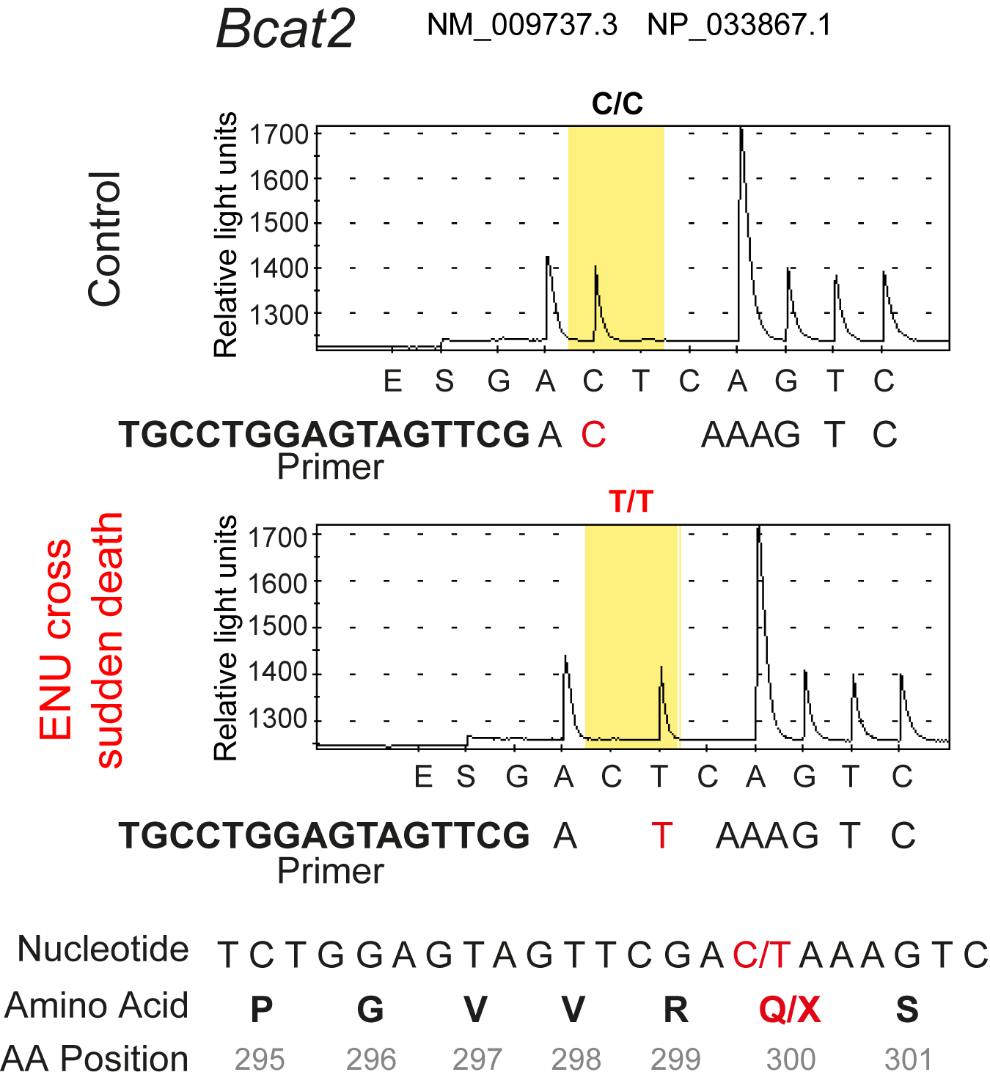
**

**Supplemental Figure S1. Validation of the *Bcat2* variant identified in the initial G3 ENU screen.** Pyrosequencing confirmation of the ENU-induced mutation in *Bcat2* showing the homozygous mutation in affected sudden death animals but absent from unaffected controls.


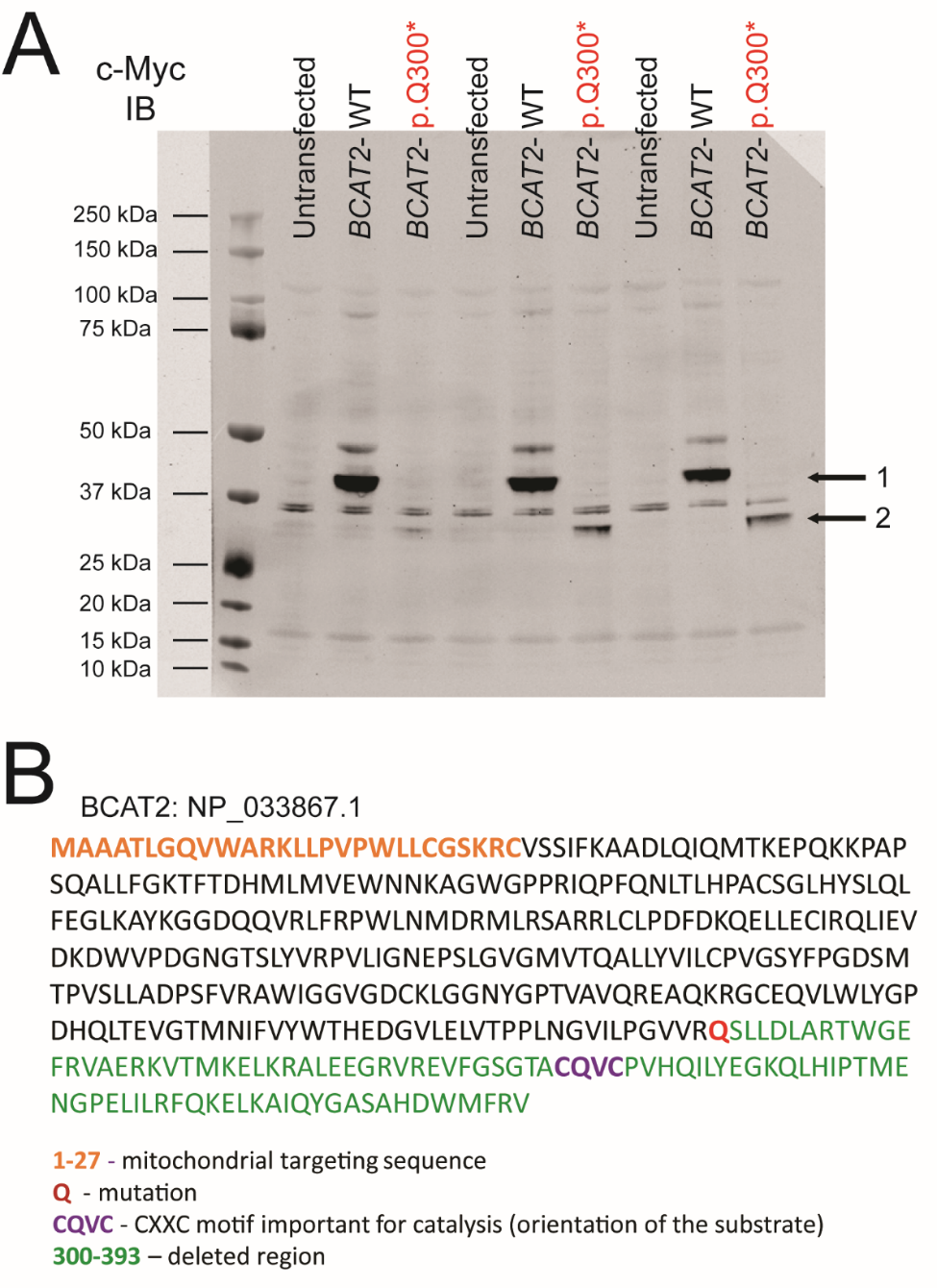


**Supplemental Figure S2. Effect of the *BCAT2* pQ300* mutation on protein translation and predicted BCAT2 protein sequence domains. (A)** *BCAT2^C1121T^* transfected in HEK293 cells results in a truncated BCAT2 protein presenting with a smaller molecular weight (arrow 2) compared to *BCAT2* WT (arrow 1). **(B)** *BCAT2^C1121T^* is predicted to lack the CXXC catalytic motif essential for enzyme catalytic activity.


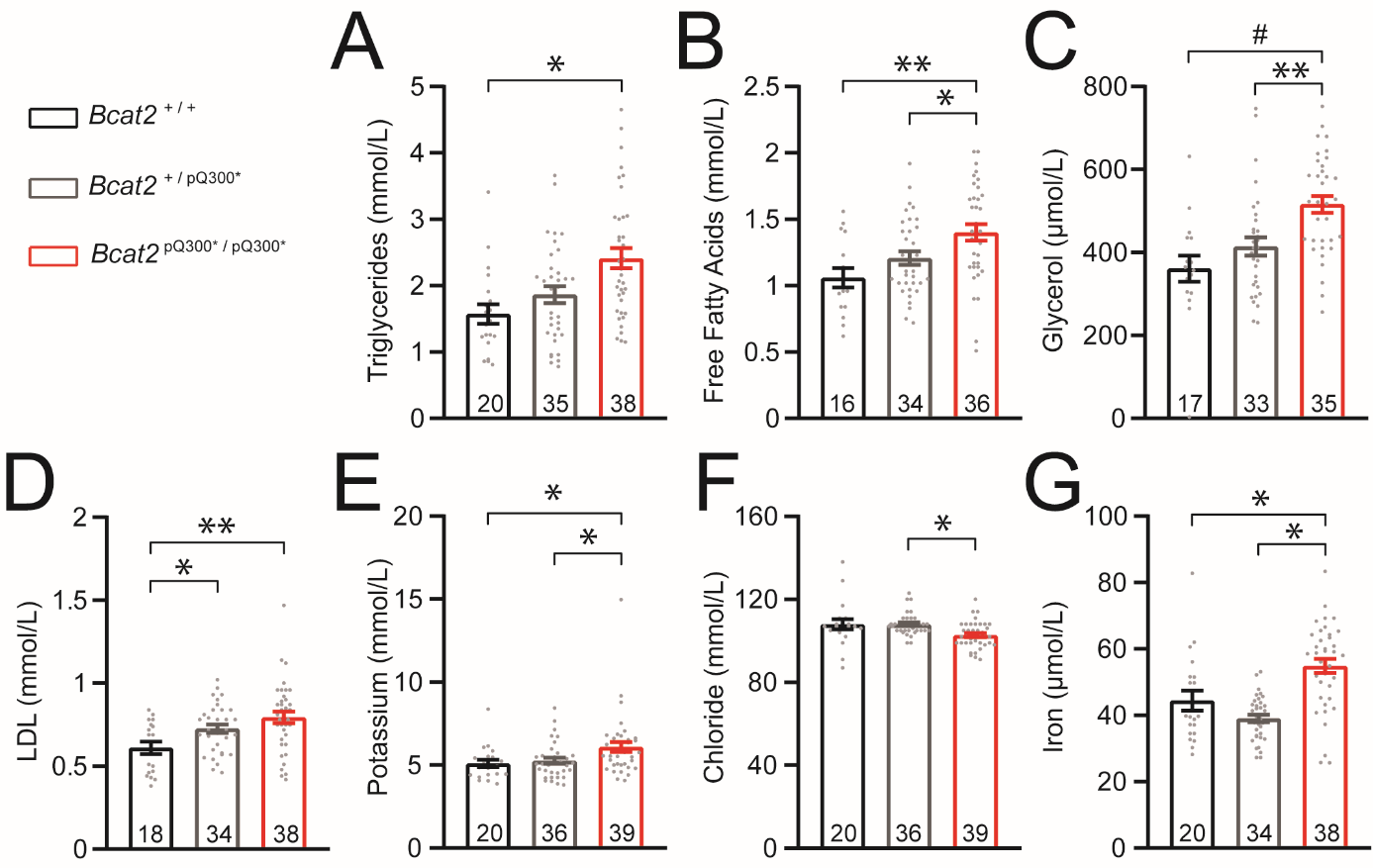


**Supplemental Figure S3. Biochemical plasmatic analyses performed in mice from batch A from the initial ENU screen.** (**A**) Triglycerides (**B**) Free fatty acids (**C**) Glycerol (**D**) Low-density lipoproteins (LDL) (**E**) Potassium (**F**) Chloride (**G**) Iron. **P* < 0.05; ***P* ≤ 0.01; #*P* ≤ 0.001.


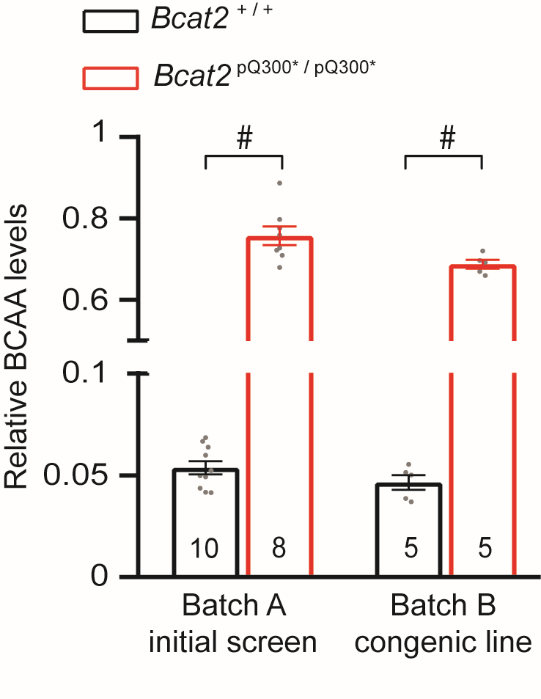


**Supplemental Figure S4. Urinary relative BCAA quantification in the initial screen (batch A) and the congenic line (batch B) of *Bcat2*^p.Q300*/p.Q300*^ mice.** Urinary BCAA levels (signal intensity from spectral region 1.08-0.90ppm), relative to signal intensity from spectral region 3.50-1.08ppm, from batch A (initial ENU screen) and batch B (backcrossed congenic line). #*P* ≤ 0.001. The number of mice per grouping is summarised on the plot.


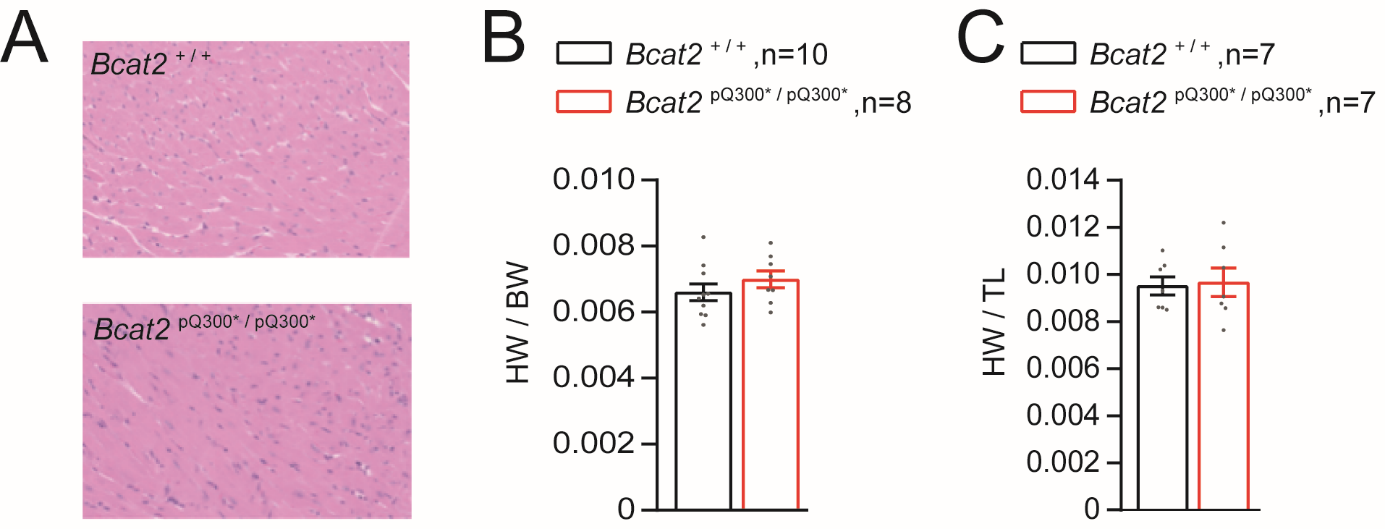


**Supplemental Figure S5. Cardiac structural characterization of *Bcat2*^p.Q300*/p.Q300*^ mice. (A)** Absence of fibrosis in hearts from *Bcat2*^p.Q300*/p.Q300*^ mice; (**B**) Unchanged heart weight to body weight ratio and **(C)** heart weight to tibia length ratio in *Bcat2*^p.Q300*/p.Q300*^ mice.


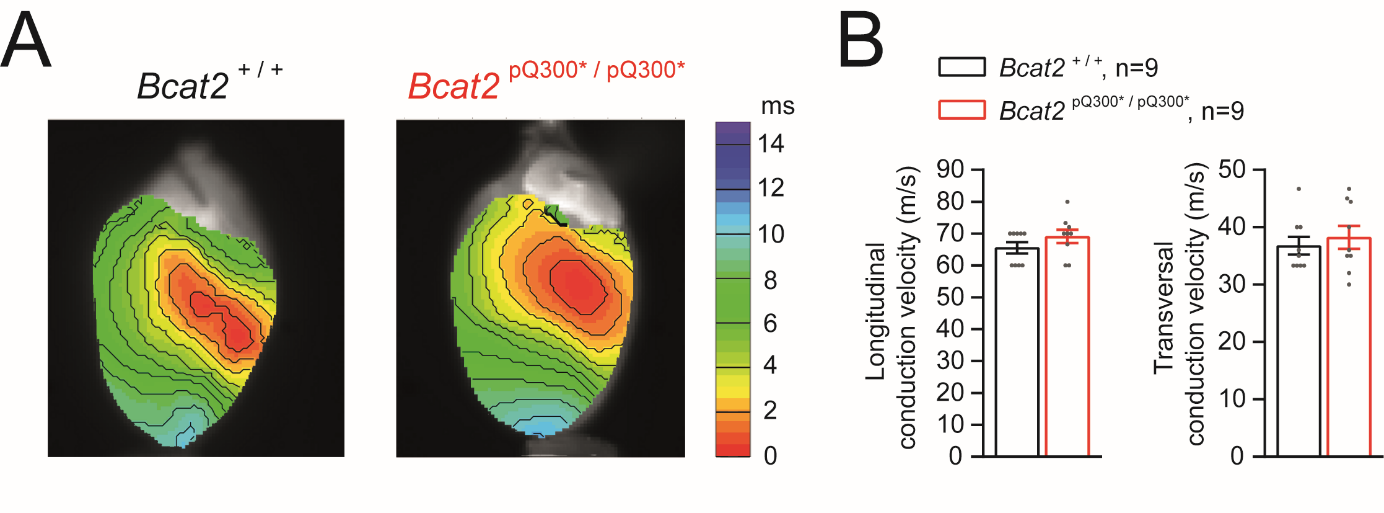


**Supplemental Figure S6. Ventricular conduction velocity is unaffected in *Bcat2*^p.Q300*/p.Q300*^ hearts.** (**A)** Typical examples of ventricular activation maps obtained with optical mapping on Langendorff perfused hearts. **(B)** Average values for longitudinal and transversal conduction velocities.


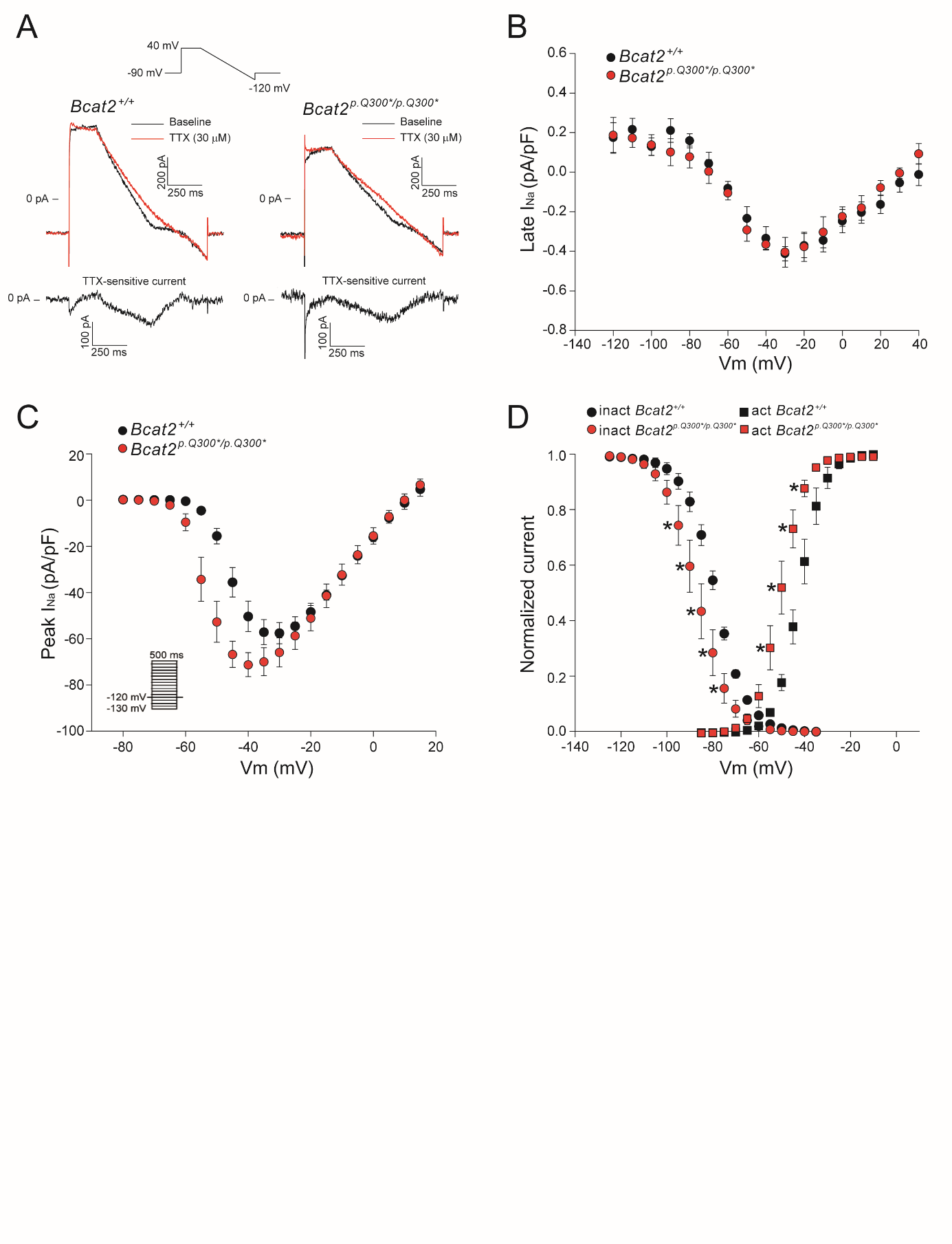


**Supplemental Figure S7.** (**A**) Representative late sodium current (I_Na_) traces recorded during a ramp protocol (inset) at baseline and after application of 30 μM tetrodotoxin (TTX). TTX -sensitive current was obtained by subtraction of the current recorded in the presence of TTX from the current recorded in the absence of the compound. (**B**) Average current-voltage (I-V) relationships for late I_Na_ measured as TTX- sensitive current shows no significant differences between *Bcat2*^+/+^ (n=8) and *Bcat2*^p.Q300*/p.Q300*^ (n=6) cardiomyocytes. (**C**) Average current-voltage (I-V) relationships for peak I_Na_ (protocol in inset) shows no significant differences between *Bcat2*^+/+^ (n=4) and *Bcat2*^p.Q300*/p.Q300*^ (n=5) cardiomyocytes. (**D**) Voltage dependence of activation and inactivation (*p<0.05). Average peak current densities and values for steady-state activation and inactivation are listed in Supplemental Table S4.

*
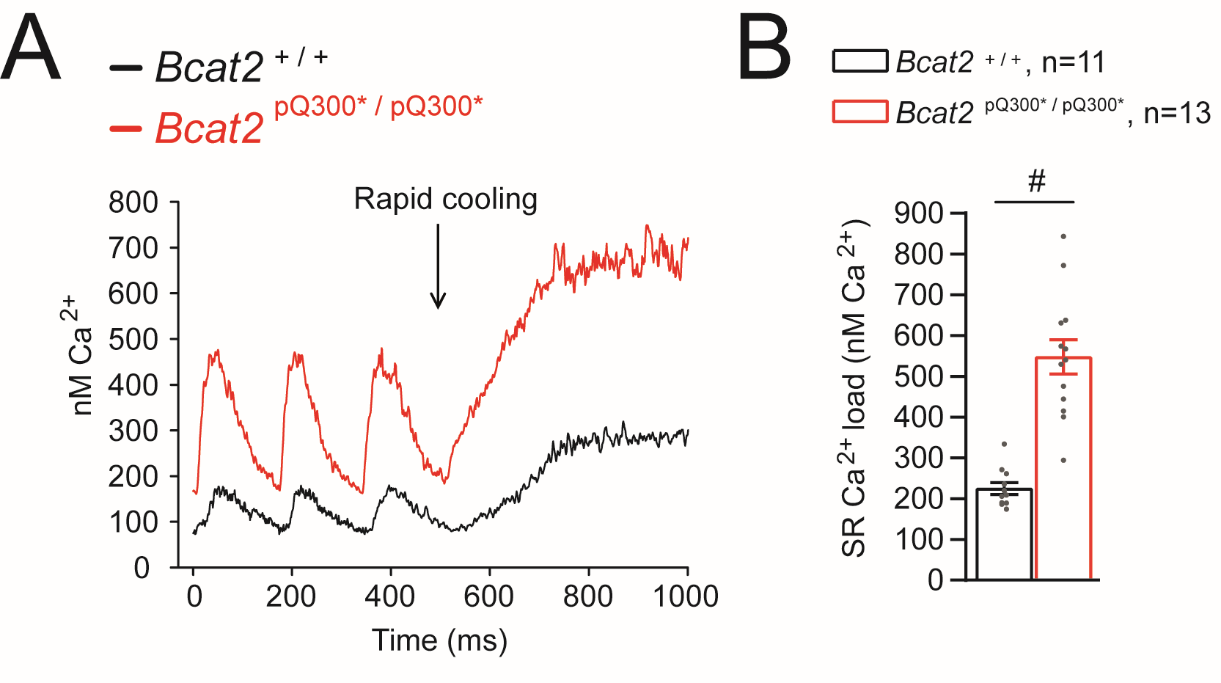
***Supplemental Figure S8. Sarcoplasmic reticulum Ca^2+^ content and fast pacing induced Ca^2+^ transients in *Bcat2*^p.Q300*/p.Q300*^ mice. (A)** Typical example of intracellular calcium recording after fast cooling enabling sarcoplasmic reticulum calcium content quantification triggered by rapid cooling in *Bcat2*^p.Q300*/p.Q300*^ mice. **(B)** Average values of sarcoplasmic reticulum calcium content for both *Bcat2*^+/+^ (n=11 cells from 6 mice) and *Bcat2*^p.Q300*/p.Q300*^ (n=11 cells from 6 mice). #*P* ≤ 0.001.


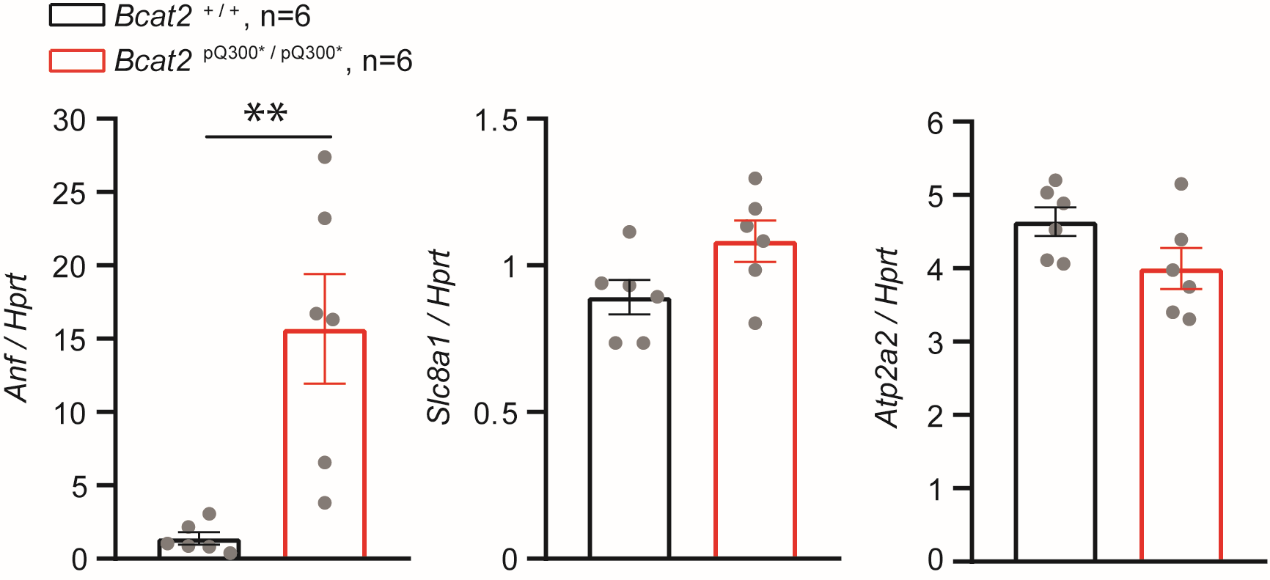


**Supplemental Figure S9.** mRNA expression levels in ventricular tissue of *Bcat2^+/+^* and *Bcat2*^p.Q300*/p.Q300*^ mice of *Anf*, *Slc8a1* and *Atp2a2* (relative to *Hprt*).


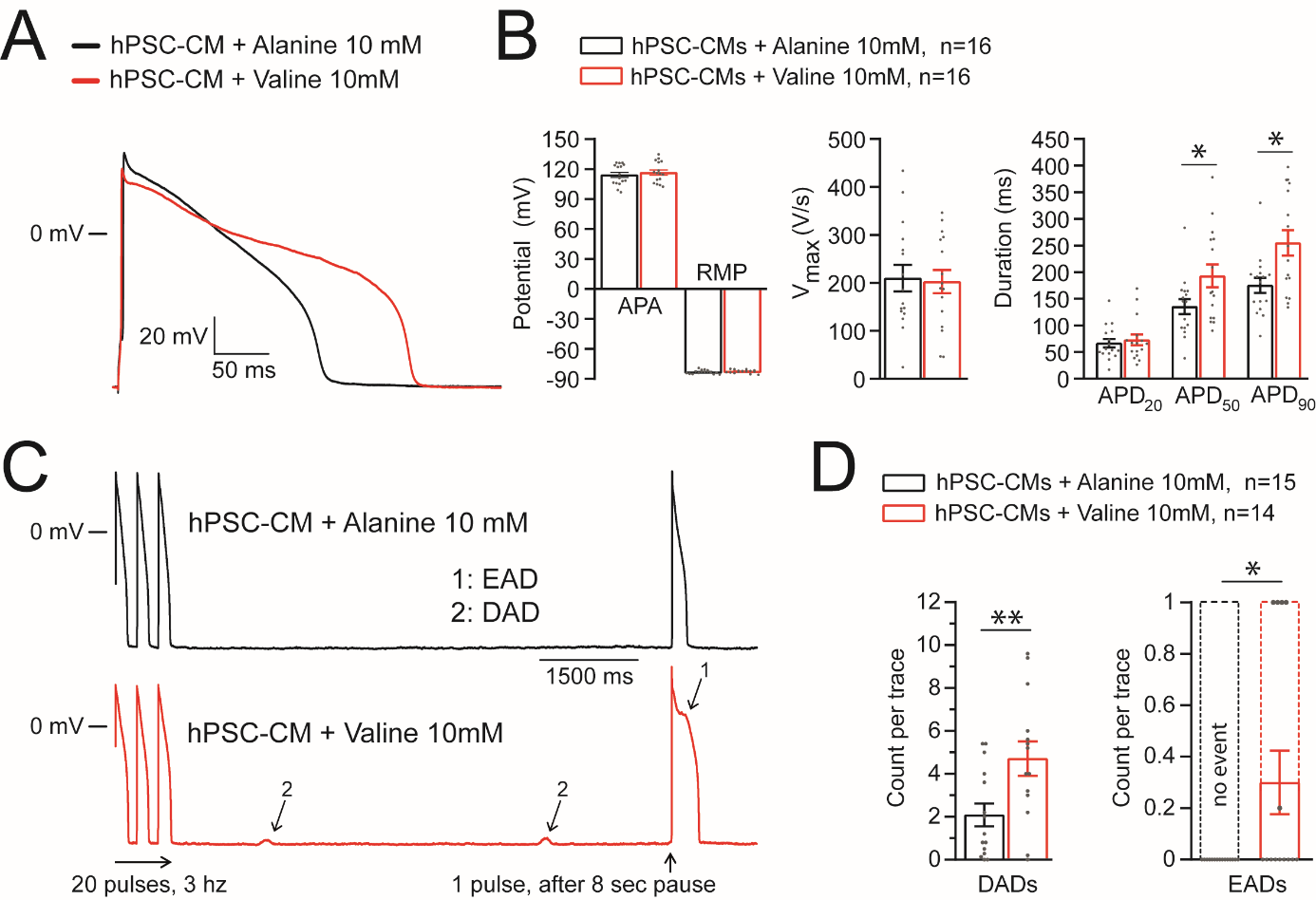
**Supplemental Figure S10. Pro-arrhythmic effects of valine (10 mM) compared to alanine (10 mM) in human PSC derived cardiomyocytes (hPSC-CMs). (A)** Typical example of APs elicited at the stimulation frequency of 1Hz from hPSC-CMs incubated with medium containing 10 mM of alanine or with 10 mM valine. **(B)** Average values for APA (action potential amplitude), RMP (resting membrane potential), V_max_ (upstroke velocity) and APD (action potential duration) at 20%, 50% and 90% repolarization (APD_20_, APD_50_, APD_90_) of hPSC-CMs incubated with either 10 mM alanine medium (n=16), or 10 mM valine medium (n=16). **(C)** Typical examples of EADs (1) and DADs (2) recorded after a fast pacing stimulation protocol (20 pulses at 3-Hz followed by a 8s pause and 1 pulse followed by a 1s pause). **(D)** Average count per trace for DADs and EADs observed in hPSC-CMs incubated with 10 mM alanine (n=15) and 10 mM valine (n=14) medium. **P* < 0.05; ***P* ≤ 0.01.

**
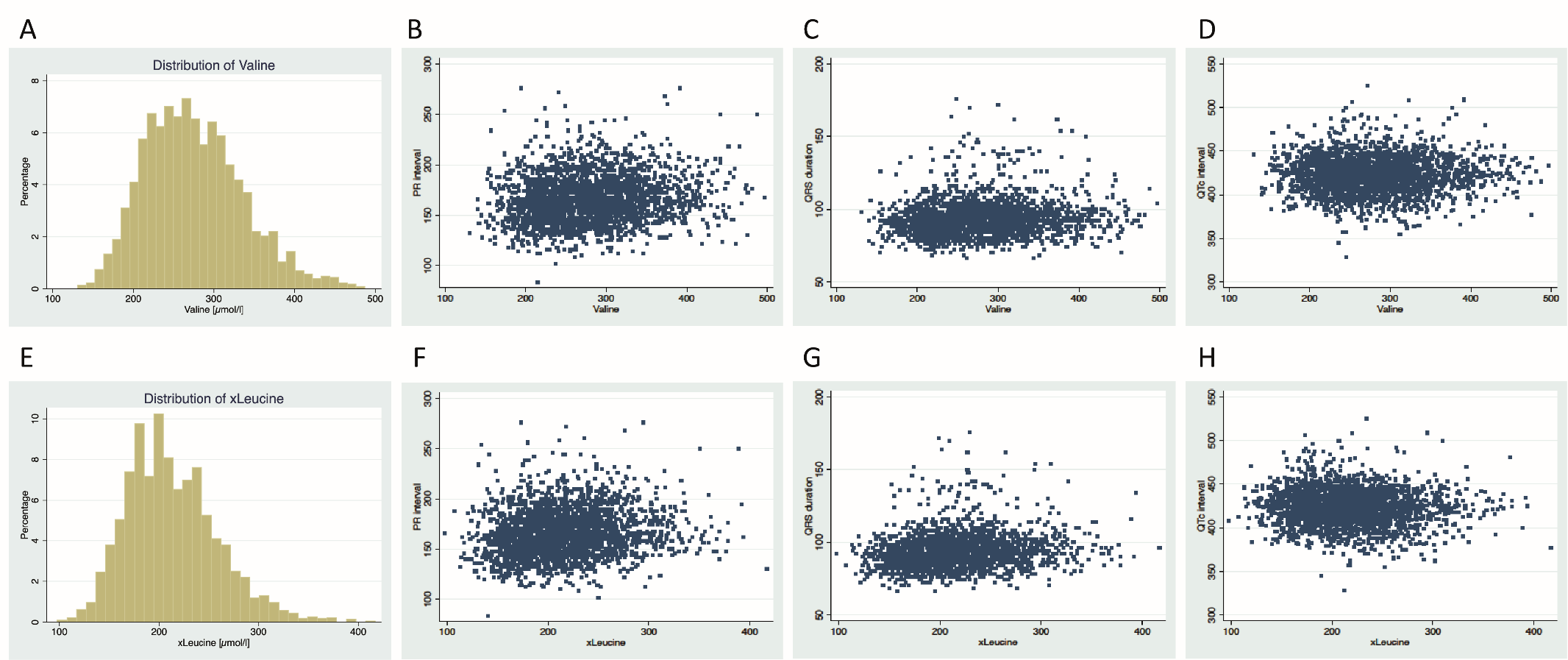
**

**Supplemental Figure 11.** The distribution of BCAAs in the KORA F4 cohort and their relation with ECG measures. The upper row shows valine results, the lower row shows xleucine results. Panels A and E, respectively, visualize the distribution of valine and xleucine, respectively. The remaining panels indicate a linear relation of the PR interval (panels B and F), the QRS duration (panels C and G), and the QTc interval (panels D and H) with valine and xleucine, respectively. The PR, QRS and QTc interval values are expressed in ms. BCAA concentrations are expressed in µmol/L.

**Supplementary Table S1:** List of parameters measured in the plasma clinical chemistry screen.

| **Parameter** | **Units** |  |  | **Parameter** | **Units** |
| --- | --- | --- | --- | --- | --- |
| Sodium | (mmol/L) |  |  | LDL | (mmol/L) |
| Potassium | (mmol/L) |  |  | Glucose | (mmol/L) |
| Chloride | (mmol/L) |  |  | Triglycerides | (mmol/L) |
| Urea | (mmol/L) |  |  | Glycerol | (µmol/L) |
| Creatinine | (µmol/L) |  |  | Free Fatty Acids | (mmol/L) |
| Calcium | (mmol/L) |  |  | Total Billirubin | (µmol/L) |
| Inorganic Phosphorus | (mmol/L) |  |  | LDH | (U/L) |
| ALP | (U/L) |  |  | Iron | (µmol/L) |
| ALT | (U/L) |  |  | Amylase | (U/L) |
| AST | (U/L) |  |  | Creatine Kinase | (U/L) |
| Total Protein | (g/L) |  |  | Uric Acid | (µmol/L) |
| Albumin | (g/L) |  |  | Ketone Bodies | (mmol/L) |
| Total Cholesterol | (mmol/L) |  |  | Fructose | (µmol/L) |
| HDL | (mmol/L) |  |  | Magnesium | (mmol/L) |

**Supplemental Table S2**: Values and statistics of measurements from *Bcat2*^+/+^ and *Bcat2* ^pQ300*/pQ300*^ mice and hearts.

|  | n | *Bcat2* ^+/+^ | n | *Bcat2* ^p.Q300*/p.Q300*^ | Statistical test | P value |
| --- | --- | --- | --- | --- | --- | --- |
| HW / BW | 10 | 0.0066 ± 0.0003 | 8 | 0.0069 ± 0.0002 | t-test | P = 0.300 |
| HW / TL | 7 | 0.0095 ± 0.0006 | 7 | 0.0096 ± 0.0004 | t-test | P = 0.838 |
| qPCR *Anf / Hprt* | 6 | 1.39 ± 0.41 | 6 | 15.67 ± 3.74 | Mann-Whitney Rank sum test | P = 0.002 |
| qPCR *Slc8a1 / Hprt* | 6 | 0.89 ± 0.06 | 6 | 1.08 ± 0.08 | t-test | P = 0.063 |
| qPCR *Atp2a2 / Hprt* | 6 | 4.64 ± 0.2 | 6 | 4 ± 0.28 | t-test | P = 0.091 |
| mTOR total (relative to GAPDH) | 7 | 1 ± 0.07 | 6 | 1.79 ± 0.33 | Mann-Whitney Rank sum test | P = 0.035 |
| P-mTOR (relative to GAPDH) | 7 | 1 ± 0.15 | 6 | 4.06 ± 0.92 | Mann-Whitney Rank sum test | P = 0.002 |
| Ratio p-mTOR/mTOR total | 7 | 1 ± 0.14 | 6 | 2.62 ± 0.61 | t-test | P = 0.017 |
| Heart rate (beat/min) | 11 | 491.6 ± 14.1 | 15 | 505.1 ± 9.7 | t-test | P = 0.420 |
| QRS interval (ms) | 11 | 10.5 ± 0.2 | 15 | 11.2 ± 0.1 | t-test | P = 0.121 |
| PR interval (ms) | 11 | 35.3 ± 0.9 | 15 | 38.7 ± 0.4 | t-test | P ≤ 0.001 |
| QT interval (ms) | 11 | 44.8 ± 1.2 | 15 | 48.7 ± 1.1 | t-test | P ≤ 0.05 |
| QTc interval (ms) | 11 | 40.4 ± 0.8 | 15 | 44.6 ± 0.8 | t-test | P ≤ 0.001 |
| Transversal CV (m/s) | 9 | 37.8 ± 1.5 | 9 | 38.2 ± 2.0 | t-test | P = 0.573 |
| Longitudinal CV (m/s) | 9 | 65.6 ± 1.8 | 9 | 69.1 ± 2.1 | t-test | P = 0.213 |
| APD_70_ (optical) (ms) | 7 | 43.9 ± 3.1 | 7 | 60.4 ± 6.0 | Mann-Whitney Rank sum test | P = 0.038 |
| Longest minus shortest APD_70_ (optical) (ms) | 7 | 9.1 ± 1.3 | 7 | 16.3 ± 1.9 | t-test | P = 0.013 |

**Supplemental Table S3**: Plasma clinical chemistry results and statistics in *Bcat2* ^+/+^ and *Bcat2* ^pQ300*/pQ300*^ mice.

| Murine model | n | *Bcat2* ^+/+^ | n | *Bcat2 ^+^*^/p.Q300*^ | n | *Bcat2* ^p.Q300*/p.Q300*^ | Statistical test and Pvalue |
| --- | --- | --- | --- | --- | --- | --- | --- |
| Triglyceride  (mmol/L) | 20 | 1.57 ± 0.14 | 35 | 1.86 ± 0.12 | 38 | 2.41 ± 0.15 | 1 way anova on ranks – Dunn’s method  - WT vs Homozygous:  P value <0.05 |
| Free fatty acids  (mmol/L) | 16 | 1.05 ± 0.07 | 34 | 1.20 ± 0.05 | 36 | 1.40 ± 0.06 | Student-Newman-Keuls Method  - WT vs Homozygous:  P value <0.05 (0.003)  -Heterozygous vs homozygous:  P value <0.05 (0.016) |
| Glycerol  (µmol/L) | 17 | 360.9 ± 31.2 | 33 | 414.3 ± 21.7 | 35 | 516.1 ± 20.4 | Student-Newman-Keuls Method  - WT vs Homozygous:  P value <0.001  -Heterozygous vs homozygous:  P < 0.001 |
| LDL  (mmol/L) | 20 | 0.61 ±0.04 | 35 | 0.72 ± 0.02 | 39 | 0.79 ± 0.03 | Student-Newman-Keuls Method  - WT vs Heterozygous:  P <0.05 (0.029)  - WT vs Homozygous:  P <0.01 (0.002) |
| Potassium  (mmol/L) | 20 | 5.1 ± 0.2 | 36 | 5.3 ± 0.2 | 39 | 6.1 ± 0.3 | 1 way anova on ranks – Dunn’s method  - WT vs Homozygous:  P <0.05  Homozygous vs heterozygous:  P <0.05 |
| Chloride  (mmol/L) | 20 | 107.9 ± 2.5 | 36 | 107.9± 0.9 | 39 | 102.7 ± 1.0 | 1 way anova on ranks – Dunn’s method  - Heterozygous vs homozygous:  P <0.05 |
| Iron  (µmol/L) | 20 | 44.4 ± 3.0 | 34 | 39.1 ± 1.2 | 38 | 54.9 ± 2.1 | 1 way anova on ranks – Dunn’s method  - WT vs homozygous:  P value <0.05  - Heterozygous vs homozygous:  P <0.05 |
| BCAAs (mmol/L) | 11 | 1.83 ± 0.1 | 9 | 2.05 ± 0.2 | 7 | 19.6 ± 2.8 | 1 way anova on ranks – Dunn’s method  - WT vs homozygous:  P <0.05  - Heterozygous vs homozygous:  P <0.05 |
| Valine (mmol/L) | 11 | 0.55 ± 0.03 | 9 | 0.68 ± 0.03 | 7 | 9.9 ± 0.54 | 1 way anova on ranks – Dunn’s method  - WT vs homozygous:  P <0.05 |
| xLeucine (mmol/L) | 11 | 1.6 ± 0.09 | 9 | 1.7 ± 0.06 | 7 | 10.2 ± 0.49 | 1 way anova on ranks – Dunn’s method  - WT vs homozygous:  P <0.05  - Heterozygous vs homozygous:  P <0.05 |

**Supplemental Table S4.** Values and statistics of patch clamp measurements in isolated cardiomyocytes from *Bcat2*^+/+^ and *Bcat2* ^pQ300*/pQ300*^ mice.

|  | n | *Bcat2* ^+/+^ | n | *Bcat2* ^p.Q300*/p.Q300*^ | Statistical test | P value |
| --- | --- | --- | --- | --- | --- | --- |
| *Action potentials* |  |  |  |  |  |  |
| APA (mV) | 13 | 112.4± 1.8 | 7 | 117.2 ±1.4 | t-test | P = 0.093 |
| RMP (mV) | 13 | -85.9 ± 0.6 | 7 | -85.5 ± 0.7 | t-test | P = 0.713 |
| V_max_ (V/s) | 13 | 509.5 ± 42.9 | 7 | 654.0 ± 39.9 | t-test | P = 0.041 |
| APD_20_ | 13 | 0.83 ± 0.08 | 7 | 0.86 ± 0.15 | t-test | P = 0.856 |
| APD_50_ | 13 | 2.29 ± 0.31 | 7 | 2.95 ± 0.78 | Mann-Whitney Rank sum test | P = 0.905 |
| APD_90_ | 13 | 88.5 ± 5.8 | 7 | 116.8 ± 8.7 | t-test | P = 0.01 |
| *Sodium current (I*_Na_) |  |  |  |  |  |  |
| Peak *I*_Na_ density at  -20 mV (pA/pF) | 4 | -48.4 ± 3.7 | 5 | -51.1 ± 5.5 | t-test | P = 0.706 |
| Steady-state activation V_1/2_ (mV) | 4 | -42.7 ± 2.1 | 5 | -50.2 ± 1.9 | t-test | P = 0.036 |
| Steady-state activation *k* (mV) | 4 | 4.8 ± 0.3 | 5 | 4.5 ± 0.2 | t-test | P = 0.413 |
| Steady-state inactivation V_1/2_ (mV) | 4 | -79.4 ± 1.0 | 5 | -86.4 ± 2.7 | t-test | P = 0.059 |
| Steady-state inactivation *k* (mV) | 4 | 6.9 ± 0.4 | 5 | 6.3 ± 0.1 | t-test | P = 0.186 |
| Late *I*_Na_ density at  -20 mV (pA/pF) | 8 | -0.37 ± 0.06 | 6 | -0.38 ± 0.06 | t-test | P=NS |

APA (action potential amplitude); RMP (resting membrane potential); *V*_max_ (upstroke velocity); APD (action potential duration) at 20%, 50% and 90% repolarization (APD_20_, APD_50_, APD_90_); *V*_1/2_ (membrane potential for the half-maximal (in)activation); k (slope factor).

**Supplemental Table S5.** Values and statistics of intracellular calcium measurements in isolated cardiomyocytes from *Bcat2*^+/+^ and *Bcat2* ^pQ300*/pQ300*^ mice.

|  | n | *Bcat2* ^+/+^ | n | *Bcat2* ^p.Q300*/p.Q300*^ | Statistical test | P value |
| --- | --- | --- | --- | --- | --- | --- |
| Diastolic Ca^2+^ (nmol/L) | 12 | 75.3 ± 8.5 | 17 | 162.8 ± 12 | t-test | P ≤ 0.001 |
| Peak Ca^2+^ transient (nmol/L) | 12 | 171.3 ± 15.5 | 17 | 468.6 ± 48.1 | Mann-Whitney Rank sum test | P ≤ 0.001 |
| Ca^2+^ transient amplitude (nmol/L) | 12 | 96.0 ± 11.6 | 17 | 305.8 ± 39.6 | Mann-Whitney Rank sum test | P ≤ 0.001 |
| Ca^2+^ transient decay (ms) | 9 | 73.7 ± 7.9 | 12 | 71.3 ± 6.6 | t-test | P = 0.813 |
| Diastolic Ca^2+^ (nmol/L) -Noradrenaline (50 nmol/L) | 9 | 110.0 ± 18.8 | 14 | 142.0 ± 18.7 | t-test | P = 0.258 |
| Systolic Ca^2+^ (nmol/L) - Noradrenaline (50 nmol/L) | 9 | 424.2 ± 62.6 | 14 | 584.3 ± 126.3 | Mann-Whitney Rank sum test | P = 0.474 |
| Ca^2+^ transient amplitude (nmol/L) - Noradrenaline (50 nmol/L) | 9 | 314.2 ± 47.5 | 14 | 442.3 ± 113.4 | Mann-Whitney Rank sum test | P = 0.633 |
| Ca^2+^ transient decay (ms) – Noradrenaline (50 nmol/L) | 9 | 47.8 ± 4.2 | 13 | 44.7 ± 2.1 | t-test | P = 0.479 |
| Diastolic Ca^2+^ (nM) following rapid cooling | 11 | 62.7 ± 6.3 | 13 | 153.4 ± 13.0 | t-test` | P ≤ 0.001 |
| Peak Ca^2+^ (nM) following rapid cooling | 11 | 287.7 ± 16.3 | 13 | 701.5 ± 47.3 | Mann-Whitney Rank sum test | P ≤ 0.001 |
| SR Ca^2+^ load (nM) following rapid cooling | 11 | 225.0 ± 14.4 | 13 | 548.0 ± 41.9 | Mann-Whitney Rank sum test | P ≤ 0.001 |
| Ratio diastolic/peak Ca^2+^ following rapid cooling | 11 | 4.9 ± 0.4 | 13 | 4.9 ± 0.4 | t-test | P = 0.994 |
| Non-triggered AP Ca^2+^ after-transients (count/trace) | 9 | 1.9 ± 0.8 | 15 | 4.3 ± 0.9 | Mann-Whitney Rank sum test | P = 0.064 |
| Triggered AP Ca^2+^ after-transients (count/trace) | 9 | 0.4 ± 0.3 | 15 | 4.6 ± 0.6 | Mann-Whitney Rank sum test | P = 0.393 |
| Total Ca^2+^ after-transients (count/trace) | 9 | 2.3 ± 0.7 | 15 | 5.5 ± 1.0 | t-test | P = 0.034 |
| Non-triggered AP Ca^2+^ after-transients (count/trace) – Noradrenaline (50 nmol/L) | 9 | 4.0 ± 0.8 | 10 | 4.8 ± 1.3 | t-test | P = 0.611 |
| Triggered AP Ca^2+^ after-transients (count/trace) – Noradrenaline (50 nmol/L) | 9 | 0.6 ± 0.6 | 10 | 6.6 ± 3.0 | Mann-Whitney Rank sum test | P = 0.012 |
| Total Ca^2+^ after-transients (count/trace) – Noradrenaline (50 nmol/L) | 9 | 4.6 ± 0.7 | 10 | 11.4 ± 3.7 | Mann-Whitney Rank sum test | P = 0.004 |

**Supplemental Table S6**: Effect of BCAAs and combination of BCAAs and rapamycin 500 nmol/L on hPSC-CM electrophysiological properties

| hPSC-CMs | n | CTL medium | n | BCAAs | n | BCAAs + Rapamycin | Statistical test  P value |
| --- | --- | --- | --- | --- | --- | --- | --- |
| RMP (mV) | 25 | -83.04 ± 0.35 | 25 | -83.19 ± 0.38 | 24 | -82.93 ± 0.47 | 1-way ANOVA on ranks – Dunn’s method  NS (Ranks) |
| APA (mV) | 25 | 111.51 ± 2.02 | 25 | 115.26 ± 1.39 | 24 | 106.67 ± 2.01 | 1-way ANOVA on ranks – Dunn’s method  NS |
| V_max_ (V/s) | 25 | 170.27 ± 28.22 | 25 | 184.96 ± 23.81 | 24 | 173.23 ± 16.45 | 1-way ANOVA NS |
| APD_20_ (ms) | 25 | 69.12 ±0.7 | 25 | 71.73 ± 5.74 | 24 | 52.82 ± 6.87 | 1-way ANOVA NS |
| APD_50_ (ms) | 25 | 143.68 ± 12.1 | 25 | 191.1 ± 21.06 | 24 | 141.42 ± 15.74 | 1-way ANOVA on ranks – Dunn’s method  NS |
| APD_90_ (ms) | 25 | 190.75 ± 14.16 | 25 | 256.51 ± 21.71 | 24 | 199.15 ± 16.46 | Student-Newman-Keuls Method  -- CTL vs BCAAs: P <0.05  - BCAAs vs BCAAs+ rapa: P <0.05 |
| DADs  (Count per trace) | 23 | 2.7 ± 0.63 | 24 | 5.37 ± 0.59 | 19 | 2.66 ± 0.57 | 1-way ANOVA on ranks – Dunn’s method  - CTL vs BCAAs: P <0.05  - BCAAs vs BCAAs+ rapa: P <0.05 |
| EADs  (Count per trace) | 23 | 0 | 24 | 0.38 ± 0.1 | 19 | 0 | 1-way ANOVA on ranks – Dunn’s method  -CTL vs BCAAs: P <0.05  - BCAAs vs BCAAs + rapa: P <0.05 |
| Diastolic Ca^2+^ (nM) | 22 | 73.62 ± 7.96 | 24 | 147.53 ± 16.47 | 22 | 93.98 ± 11.86 | 1-way ANOVA on ranks – Dunn’s method  -CTL vs BCAAs: P <0.05  - BCAAs vs BCAAs + rapa: P <0.05 |
| Peak transient (nM) | 22 | 182.64 ± 15.42 | 24 | 312.67 ± 24.29 | 22 | 187.29 ± 21.48 | 1-way ANOVA on ranks – Dunn’s method  -CTL vs BCAAs: P <0.001  - BCAAs vs BCAAs + rapa: P <0.001 |
| Transient amplitude (nM) | 22 | 105.29 ± 10.54 | 24 | 166.93 ± 11.48 | 22 | 93.31 ± 14.48 | 1-way ANOVA on ranks – Dunn’s method  -CTL vs BCAAs: P <0.05  - BCAAs vs BCAAs + rapa: P <0.05 |
| Non-triggered AP Ca^2+^ transient (EC/T) | 17 | 1 ± 0.4 | 16 | 4 ± 1.39 | 14 | 1 ± 0.72 | 1-way ANOVA on ranks  NS |
| Triggered AP Ca^2+^ transient (EC/T) | 17 | 1 ± 0.27 | 16 | 2 ± 0.71 | 14 | 1 ± 0.37 | 1-way ANOVA on ranks  NS |
| Total Ca^2+^ transient (EC/T) | 17 | 2 ± 0.41 | 16 | 6 ± 1.32 | 14 | 2 ± 0.76 | 1-way ANOVA on ranks – Dunn’s method  -CTL vs BCAAs: P <0.05  - BCAAs vs BCAAs + rapa: P <0.05 |

APA (action potential amplitude); RMP (resting membrane potential); *V*_max_ (upstroke velocity); APD (action potential duration) at 20%, 50% and 90% repolarization (APD_20_, APD_50_, APD_90_); EADs (early afterdepolarizations); DADs (delayed afterdepolarizations).

**Supplemental Table S7**: Effect of rapamycin 500 nmol/L on hPSC-CMs electrophysiological properties.

| hPSC-CMs | n | CTL medium | n | CTL medium + Rapamycin | Statistical test |
| --- | --- | --- | --- | --- | --- |
| RMP (mV) | 25 | -83.04 ± 0.35 | 11 | -83.50 ± 0.93 | Mann-Whitney Rank sum test  NS |
| APA (mV) | 25 | 111.51 ± 2.02 | 11 | 108.38 ± 2.84 | Mann-Whitney Rank sum test  NS |
| V_max_ (V/s) | 25 | 170.27 ± 28.22 | 11 | 190.07 ± 23.23 | t-test  NS |
| APD_20_ (ms) | 25 | 69.12 ±0.7 | 11 | 77.19 ± 12.63 | t-test  NS |
| APD_50_ (ms) | 25 | 143.68 ± 12.1 | 11 | 155.17 ± 22.63 | t-test  NS |
| APD_90_ (ms) | 25 | 190.75 ± 14.16 | 11 | 204.21 ± 20.2 | t-test  NS |
| DADs  (Count per trace) | 23 | 2.7 ± 0.63 | 9 | 0.99 ± 0.34 | Mann-Whitney Rank sum test  NS |
| EADs  (Count per trace) | 23 | 0 | 9 | 0 | Not tested  No events recorded |

APA (action potential amplitude); RMP (resting membrane potential); *V*_max_ (upstroke velocity); APD (action potential duration) at 20%, 50% and 90% repolarization (APD_20_, APD_50_, APD_90_); EADs (early afterdepolarizations); DADs (delayed afterdepolarizations).

**Supplementary Table S8**. Effect of valine 10 mmol/l compared to alanine 10 mmol/l on hPSC-CMs electrophysiological properties.

| hPSC-CMs | n | Alanine 10mM | n | Valine 10mM | Statistical test |
| --- | --- | --- | --- | --- | --- |
| RMP (mV) | 16 | -83 ± 0.48 | 16 | -82.53 ± 0.42 | t-test  NS |
| APA (mV) | 16 | 114.26 ± 2.57 | 16 | 116.8 ± 2.6 | Mann-Whitney Rank sum test  NS |
| V_max_ (V/s) | 16 | 209.97 ± 27.56 | 16 | 202.66 ± 24.32 | t-test  NS |
| APD_20_ (ms) | 16 | 66.82 ± 8.02 | 16 | 72.88 ± 10.13 | Mann-Whitney Rank sum test  NS |
| APD_50_ (ms) | 16 | 135.46 ± 14.05 | 16 | 193.03 ± 21.54 | t-test  P = 0.033 |
| APD_90_ (ms) | 16 | 175.2 ± 14.28 | 16 | 254.89 ± 23.87 | Mann-Whitney Rank sum test  P = 0.025 |
| DADs  (Count per trace) | 15 | 2.09 ± 0.53 | 14 | 4.71 ± 0.8 | t-test  P = 0.01 |
| EADs  (Count per trace) | 15 | 0 | 14 | 0.3 ± 0.12 | Mann-Whitney Rank sum test  P= 0.014 |

APA (action potential amplitude); RMP (resting membrane potential); *V*_max_ (upstroke velocity); APD (action potential duration) at 20%, 50% and 90% repolarization (APD_20_, APD_50_, APD_90_); EADs (early afterdepolarizations); DADs (delayed afterdepolarizations).

**Supplementary Table S9**. Surface ECG parameters in male and female *Bcat2* ^+/+^ and *Bcat2* ^p.Q300*/p.Q300*^ mice.

| Murine model | n | *Bcat2* ^+/+^ | n | *Bcat2* ^p.Q300*/p.Q300*^ | Statistical test | P value |
| --- | --- | --- | --- | --- | --- | --- |
| *Males* |  |  |  |  |  |  |
| Heart rate (beat/min) | 6 | 474.6 ± 22.1 | 8 | 512.2 ± 14.0 | t-test | P = 0.129 |
| QRS interval (ms) | 6 | 10.2 ± 0.5 | 8 | 11.5 ± 0.5 | t-test | P = 0.039 |
| PR interval (ms) | 6 | 34.7 ± 1.2 | 8 | 38.1 ± 0.6 | t-test | P = 0.009 |
| QT interval (ms) | 6 | 44.5 ± 1.7 | 8 | 48.2 ± 1.6 | t-test | P = 0.120 |
| QTc interval (ms) | 6 | 39.4 ± 0.6 | 8 | 44.4 ± 1.3 | t-test | P = 0.005 |
| *Females* |  |  |  |  |  |  |
| Heart rate (beat/min) | 5 | 511.9 ± 19.2 | 7 | 497.1 ± 16.0 | t-test | P = 0.530 |
| QRS interval (ms) | 5 | 10.8 ± 0.4 | 7 | 10.8 ± 0.5 | t-test | P = 0.969 |
| PR interval (ms) | 5 | 36.0 ± 1.6 | 7 | 39.4 ± 0.6 | t-test | P = 0.037 |
| QT interval (ms) | 5 | 45.2 ± 2.4 | 7 | 49.4 ± 1.9 | t-test | P = 0.159 |
| QTc interval (ms) | 5 | 41.7 ± 1.7 | 7 | 44.8 ± 1.2 | t-test | P = 0.130 |
